# Community-Acquired Pneumonia: Disease Course Prediction With a 5-Gene Signature

**DOI:** 10.1016/j.chpulm.2026.100256

**Published:** 2026-03-10

**Authors:** Holger Kirsten, Sebastian Weis, Peter Ahnert, Martin Witzenrath, Brendon P. Scicluna, Knut Krohn, Michael Rade, Friedemann Horn, Catharina Bertram, Kristin Reiche, Dennis Löffler, Conny Blumert, Stefan Jenner, Kai Sohn, Geraldine Nouailles, Michael Kiehntopf, Petra Creutz, Maciej Rosolowski, Markus Loeffler, Norbert Suttorp, PROGRESS Study Group, Markus Scholz, Michael Bauer

**Affiliations:** a Institute for Medical Informatics, Statistics and Epidemiology (IMISE), Leipzig University, Leipzig, Germany; b German Center for Lung Research (DZL), Associated Partner PROGRESS, Universität Leipzig, Leipzig, Germany; c Center for Scalable Data Analytics and Artificial Intelligence (ScaDS.AI), Dresden/Leipzig, Germany; d Department of Anesthesiology and Intensive Care Medicine, Jena University Hospital, Friedrich-Schiller-University Jena, Jena, Germany; e Institute for Infectious Disease and Infection Control, Jena University Hospital, Friedrich-Schiller-University Jena, Jena, Germany; f Leibniz Institute for Leibniz Institute for Natural Product Research and Infection Biology - Hans Knöll Institute, Jena, Germany; g Charité - Universitätsmedizin Berlin, corporate member of Freie Universität Berlin and Humboldt-Universität zu Berlin, Department of Infectious Diseases, Respiratory Medicine and Critical Care, Berlin, Germany; h German Center for Lung Research (DZL), Partner Site Charité, Berlin, Germany; i Centre for Molecular Medicine and Biobanking, University of Malta, Msida, Malta; j Department of Applied Biomedical Science, Faculty of Health Sciences, Mater Dei hospital, University of Malta, Msida, Malta; k Core Unit DNA Technologies, Medical Faculty, Leipzig University, Leipzig, Germany; l Department of Diagnostics, Fraunhofer Institute for Cell Therapy and Immunology, Leipzig, Germany; m Fraunhofer Institute for Interfacial Engineering and Biotechnology, Stuttgart, Germany; n Department of Clinical Chemistry and Laboratory Medicine, Jena University Hospital, Friedrich-Schiller-University Jena, Jena, Germany; o Integrated Biobank Jena (IBBJ), Jena University Hospital, Friedrich-Schiller-University Jena, Jena, Germany

**Keywords:** community-acquired pneumonia, gene expression, outcome prediction, prospective study, validation

## Abstract

**Background:**

Clinical decision-making for patients with community-acquired pneumonia (CAP) at risk of organ dysfunction and death is currently guided by clinical evaluation and scores. Every fifth patient with CAP requires admission to the ICU, with a subsequent high mortality; delayed admission to the ICU increases this risk.

**Research Question:**

Can a transcriptomic signature improve identification of at-risk patients compared with conventional scores and metrics?

**Study Design and Methods:**

Time-course transcriptomic data were obtained from blood samples taken from 455 participants in 41 centers enrolled in the Progression of Community-Acquired Pneumonia in the Hospital (PROGRESS) trial, a prospective observational cohort study of hospitalized patients with CAP who did not initially require organ support. Discovery (n = 240) and validation (n = 215) cohorts were randomly assigned. Transcriptome data were analyzed for association with a severe CAP course, defined as a composite of requirement for ICU admission or 28-day mortality. Predictive performance of the gene expression profiles was compared against clinical scores and serum markers, and validated in publicly available transcriptomic data sets.

**Results:**

A 5-gene signature consisting of *SIGLEC14*, *TNFSF14*, *YOD1*, *CLEC4A*, and *KLRB1* (STYCK) was identified and validated to predict clinical deterioration with subsequent ICU admission or 28-day mortality (AUC_discovery_, 0.82; 95% CI, 0.71-0.90; *P* = 0.00000065; AUC_validation_, 0.81; 95% CI, 0.70-0.90; *P* = 0.0000013). The signature outperformed clinical scores in predicting severe CAP and improved prediction when added to the Sequential Organ Failure Assessment score (AUC_SOFA_, 0.70 vs AUC_SOFA+STYCK_, 0.83; *P* = .0002). Prognostic value was confirmed in 6 of 17 publicly available sepsis cohorts, particularly those with low case fatality.

**Interpretation:**

We identified a 5-gene transcriptomic signature that, taken soon after hospital admission, was shown to predict disease course of hospitalized patients with CAP. STYCK was superior to conventional, currently used scores and clinical metrics in predicting this deterioration and improved, if added to the Sequential Organ Failure Assessment, its performance.

**Clinical Trial Registration:**

ClinicalTrials.gov; No.: NCT02782013; URL: www.clinicaltrials.gov

**Take-Home Points**

**Study Question:** Can a transcriptomic signature improve identification of at-risk patients compared with conventional scores and metrics?

**Results:** In hospitalized patients with CAP, we identified a 5-gene signature (STYCK) that improves the predictive value of clinical scores regarding the imminent requirement of intensive care treatment after admission.

**Interpretation:** STYCK was superior to conventional, currently used scores and clinical metrics in predicting this deterioration and improved, if added to the Sequential Organ Failure Assessment, its performance.

Pneumonia is a leading cause of morbidity and mortality. Each year, approximately 660,000 patients in Germany alone are diagnosed with community-acquired pneumonia (CAP). Almost one-half require hospitalization, and 13% die within 30 days.^1^ The course of CAP in hospitalized patients is difficult to predict, with some rapidly deteriorating to sepsis. Every fifth patient with CAP admitted to US hospitals requires admission to the ICU with a subsequent high mortality.^2^^,^^3^ Because delayed ICU admission is a well-recognized risk factor for mortality,^4^^,^^5^ early risk prediction (ie, at hospital admission) could potentially improve patient care and increase survival rates.^3^ Tools for early prediction of deterioration are scarce but could provide important benefits to clinicians and patients.^6^

The utility of existing biomarkers to predict a severe course for CAP is rather mediocre.^7^ Laboratory measurements such as C-reactive protein and IL-6 were inferior to the Infectious Diseases Society of America/American Thoracic Society criteria^8^ for predicting ICU admission. The CURB-65 score, comprising confusion, urea, respiratory rate, BP, and age ≥ 65 years, predicted 30-day mortality but failed to indicate need for ICU admission.^7^ We have previously reported that the Sequential Organ Failure Assessment (SOFA) score better reflects the severity of CAP compared with other clinical scores.^9^

Transcriptome-based multiplexed biomarkers have been described for patients with severe CAP or sepsis.^10^, ^11^, ^12^, ^13^, ^14^ However, these biomarkers were largely developed from cohorts already admitted to intensive care and are thus of limited use for prediction of a severe course in the early phase of CAP (eg, when seen in the emergency department). Molecular predictors can discriminate between CAP and non-CAP,^10^ CAP and patients with hospital-acquired pneumonia,^15^ sterile inflammation vs sepsis,^16^ the course of patients with suspected sepsis,^17^ inflammation severity in sepsis,^18^ and bacterial vs viral infection.^19^ Signatures can also serve as indicators of overall disease severity.^20^ Despite the obvious clinical need, early identification of patients with CAP at risk of a severe disease course with ICU admission and death is lacking. We thus used gene expression data from the prospective Progression of Community-Acquired Pneumonia in the Hospital (PROGRESS) study, which enrolled patients hospitalized with CAP, to identify a signature that can predict the disease course early on admission.

## Study Design and Methods

### Study Design and Population

All participants were enrolled within PROGRESS, a prospective multicenter observational cohort study recruiting patients with CAP requiring hospitalization in Germany and Austria (ClinicalTrials.gov No. NCT02782013).^21^ Patients with CAP were enrolled soon after hospital admission and after informed consent.

Data were compared from patients with and without a severe disease course, defined as a composite end point consisting of 28-day all-cause mortality (in this cohort, the 28- and 30-day mortality rates were identical) or clinical worsening (ICU admission for CAP-specific treatment). Specific criteria included a need for significant respiratory support, namely invasive or noninvasive ventilation, extracorporeal oxygenation, or oxygen supplementation ≥ 6 L/min, (except for patients with home ventilation); catecholamines; or hemodialysis (except for patients with chronic kidney disease).^9^

The study protocol was approved by the ethics committee of the University of Jena (2403-10/08), and by locally responsible Ethics Committees for each study site, in accordance with Good Clinical Practice guidelines^22^ and the provisions of the Declaration of Helsinki.^23^ The study population was randomly separated into discovery and validation cohorts (e-Fig 1).

### Sampling and Data Collection

RNA was isolated from venous blood, DNase digested, concentrated, quantified, and quality-controlled. For array-based gene expression analysis, purified RNA was hybridized to Illumina HT-12v4 Expression-BeadChips (Illumina). Low-quality samples were removed. Data were log2-transformed, quantile-normalized, batch-corrected and filtered for minimum expression levels, resulting in 26,601 transcripts representing 16,329 unique genes.

For sequencing-based expression quantification, libraries were prepared using globin-messenger RNA depleted RNA, and sequencing was performed with HiSeq2500 sequencing (Illumina), with an average sequencing depth of 100 million clusters per sample and 2× 100b paired-end reads. Data analysis included demultiplexing, trimming, filtering, removal of low-quality bases, quantification at the gene level, and quality assessment. Sequencing data were primarily used to test the robustness of the gene expression signature ensuring independence from a specific measurement technology, an important aspect for future onsite test developments.

### Statistical Analysis

For the identification of the gene signature, a mixed-effects model was applied to the full time-course data, accounting for repeated measurements of gene expression between day 0 and day 4. The mixed-effects model was adjusted for age, sex, BMI, smoking, comorbidities, SOFA score, and blood cell counts, partly inferred using CIBERSORT.^24^ The false discovery rate (FDR) and proportion of null values of all tested hypotheses (Eta_1_) were calculated with the R-package fdrtool1.2.15. Ingenuity Pathway Analysis was used for pathway enrichment and activation analysis.^25^

Signatures were identified in a discovery cohort of 240 participants. Only transcripts with a significant correlation (ρ ≥ 0.5, FDR ≤ 0.05) in the 2 measurement platforms (ie, chip arrays, next-generation sequencing), were considered. We also performed leave-1-out cross-validation using the mixed-effects model. The resulting 240 gene lists were filtered for minimum fold change, FDR, expression level, unique, not highly correlated, and robustly associating genes overlapping among the gene lists. Expression scores were then calculated from the remaining genes.^16^^,^^19^ Prediction performance of the final signature was quantified as the area under the curve (AUC) from the receiver operator characteristic analysis, using data from day of study inclusion only, to focus on the clinically realistic scenario of prediction as early as possible. CIs were based on bootstrap sampling. Resulting AUCs were used to rank optimal fold change and FDR cutoffs.

To unite each of the 240 gene lists, expression scores of genes appearing across multiple top lists were calculated, enabling selection of the signature with the optimal AUC. The sum of the scaled signature and SOFA scores was also assessed.

To assess for potential confounding, we performed 2 separate sensitivity analyses. We reran the primary fully adjusted mixed-effects model for the signature genes, adding an additional covariate for either (1) antibiotic use within 5 days before admission, or (2) the absolute lymphocyte count.

Further details are available in e-Appendix 2.

### Data Deposition

Raw data files for RNA microarray and raw counts/normalized counts of sequencing data are deposited in the EMBL-EBI BioStudies database (accession No. E-MTAB-11796), and sequencing data are listed at zenodo.org (https://doi.org/10.5281/zenodo.6574945).

## Results

### Characteristics of the Study Population

The overall PROGRESS cohort consisted of 1,798 hospitalized patients diagnosed with CAP (e-Tables 1, 2).^9^ Gene expression was measured in 1,196 whole blood samples from 545 patients, from which 10 expression arrays were excluded for quality reasons. Filtered samples comprised 1,186 gene expression data sets from 543 patients. From those, we excluded data from 88 patients who were immediately transferred to intensive care due to their CAP illness severity. We retained transcriptomic data from 43 patients with CAP immediately transferred to intensive care for nonpulmonary reasons. Hence, the study cohort consisted of 455 patients with 885 gene expression data sets that were repeatedly obtained between days 0 and 4 (e-Figs 1, 2; e-Table 3). Forty-nine patients subsequently progressed to severe CAP (ie, CAP-associated ICU admission and/or 28-day overall mortality). The 28-day mortality in this group was 30.6% (15 of 49). The remaining 406 participants formed the low illness severity control group (e-Fig 2). The total study cohort was randomly divided into discovery (n = 240) and validation (n = 215) cohorts (e-Figs 2B, 2C; Table 1, Table 2). The elapsed time between the first RNA measurement and ICU admission or death is shown in e-Figure 2D.

**Table 1:** Baseline Patient Characteristics

Characteristic	Discovery Cohort (n = 240)	Validation Cohort (n = 215)	Total (N = 455)	Missing Values
Age, y	66 (49-75)	65 (45-74)	65 (48-75)	0 (0)
Sex, male	153 (63.7)	135 (62.8)	288 (63.3)	0 (0)
Sex, female	87 (36.3)	80 (37.2)	167 (36.7)	0 (0)
BMI, kg/m^2^	26.47 (23.1-30.1)	25.83 (22.5-29.8)	26.22 (22.6-30.0)	2 (0.4)
Smoking within last year	82 (34.7)	87 (40.7)	169 (37.1)	5 (1.1)
Smoking years	12.5 (0-30)	16.5 (0-30)	15 (0-30)	51 (11.2)
Length of hospital stay	7 (5-10)	7 (5-10)	7 (5-10)	14 (3.1)
Comorbidities				
None	102 (42.5)	91 (42.3)	193 (42.4)	0 (0)
Diabetes	43 (17.9)	45 (20.9)	88 (19.3)	0 (0)
Chronic heart disease	81 (34.6)	64 (30.3)	145 (31.9)	10 (2.2)
Chronic cerebrovascular disease	13 (5.4)	9 (4.2)	22 (4.8)	2 (0.4)
Chronic kidney disease	22 (9.3)	24 (11.4)	46 (10.1)	7 (1.5)
Chronic liver disease	3 (1.3)	9 (4.2)	12 (2.6)	3 (0.7)
Chronic respiratory disease	83 (34.7)	60 (28.2)	143 (31.4)	3 (0.7)
Comorbidity score	1 (0-2)	1 (0-2)	1 (0-2)	0 (0)
Platelets, cells/μL	222 (169-289)	232 (166-293)	226 (167-292)	26 (5.7)
Total neutrophils, %	81.7 (72.7-88.0)	82.2 (73.5-87.4)	81.7 (73.1-88.0)	185 (40.7)
Lymphocytes, %	10.10 (5.95-15.25)	9.22 (6.00-15.21)	9.83 (5.96-15.29)	187 (41.1)
Pathogen^a^				
*Chlamydia pneumoniae*	3 (1.2)	1 (0.5)	4 (0.9)	
*Escherichia coli*	2 (0.8)	2 (0.9)	4 (0.9)	
*Mycoplasma pneumoniae*	3 (1.2)	3 (1.4)	6 (1.3)	
*Staphylococcus aureus*	4 (1.6)	4 (1.8)	8 (1.7)	
*Staphylococcus epidermidis*	4 (1.6)	3 (1.4)	7 (1.5)	
*Streptococcus pneumoniae*	8 (3.3)	3 (1.4)	11 (2.4)	
Other	30 (12.2)	21 (9.5)	51 (10.9)	
Tested, but no pathogen reported	47 (19.2)	47 (21.3)	94 (20.2)	
Not done	144 (58.8)	137 (62.0)	281 (60.3)	
Drugs				
Nonsteroidal antiinflammatory drug	88 (36.7)	77 (35.8)	165 (36.3)	
Antibiotics (within 5 d before admission)	28 (11.7)	38 (17.7)	66 (14.5)	
Corticosteroids	36 (15.0)	28 (13.0)	64 (14.1)	

Data are presented as No. (%) are median (interquartile range).

a Polymicrobial infection possible.

**Table 2:** Outcomes of Patients in the Discovery and Validation Cohorts

Outcome/Scores	Discovery Cohort (n = 240)	Validation Cohort (n = 215)	Total (N = 455)	Missing Values
Composite end point	25 (10.4)	24 (11.2)	49 (10.8)	0 (0)
Future ventilation	17 (7.2)	14 (6.6)	0 (0)	8 (1.8)
28-d mortality	5 (2.1)	10 (4.7)	15 (3.3)	0 (0)
Worst future SOFA score	2 (2-3)	2 (2-4)	2 (2-4)	45 (9.9)
CRB65	1 (1-2)	1 (1-2)	1 (1-2)	0 (0)
CURB65	2 (1-2)	2 (1-3)	2 (1-2)	0 (0)
ATS minor criteria	2 (1-3)	2 (1-3)	2 (1-3)	0 (0)
> 2 ATS minor criteria	86 (35.8)	76 (35.3)	162 (35.6)	0 (0)
PSI	3 (2-4)	3 (2-4)	3 (2-4)	0 (0)
SIRS	2 (2-2)	2 (2-2)	2 (2-2)	0 (0)
qSOFA	1 (0-1)	1 (0-1)	1 (0-1)	0 (0)
Halm score	2 (1-3)	2 (1-3)	2 (1-3)	0 (0)
SCAP	7.5 (5-15)	6 (0-15)	6 (0-15)	0 (0)
SMART-COP	3 (2-4)	3 (1-4)	3 (1-4)	0 (0)
Procalcitonin	0.34 (0.10-3.18)	0.22 (0.12-1.85)	0.26 (0.11-2.48)	76 (16.7)
C-reactive protein	169 (96-245)	159 (91-230)	165 (94-242)	28 (6.2)
Intensive care admission	22 (9.2)	23 (10.7)	45 (9.9)	0 (0)

Data are presented as No. (%) are median (interquartile range). ATS = American Thoracic Society; CRB65 = similar to CURB but additionally considering ≥ 65 y of age; CURB65 = confusion, urea, respiratory rate, blood pressure and age ≥ 65; PSI = Pneumonia Severity Index, qSOFA = quick Sequential Organ Failure Assessment; SCAP = severe community acquired pneumonia score; SIRS = systemic inflammatory response syndrome; SMART-COP = systolic BP, multilobar chest radiographic involvement, low albumin level, high respiratory rate, tachycardia, confusion, poor oxygenation, and low arterial pH score; SOFA = Sequential Organ Failure Assessment.

### Five-Gene Signature Consisting of *SIGLEC14*, *TNFSF14*, *YOD1*, *CLEC4A*, and *KLRB1* Predicts Severe CAP Courses

**Figure 1:**
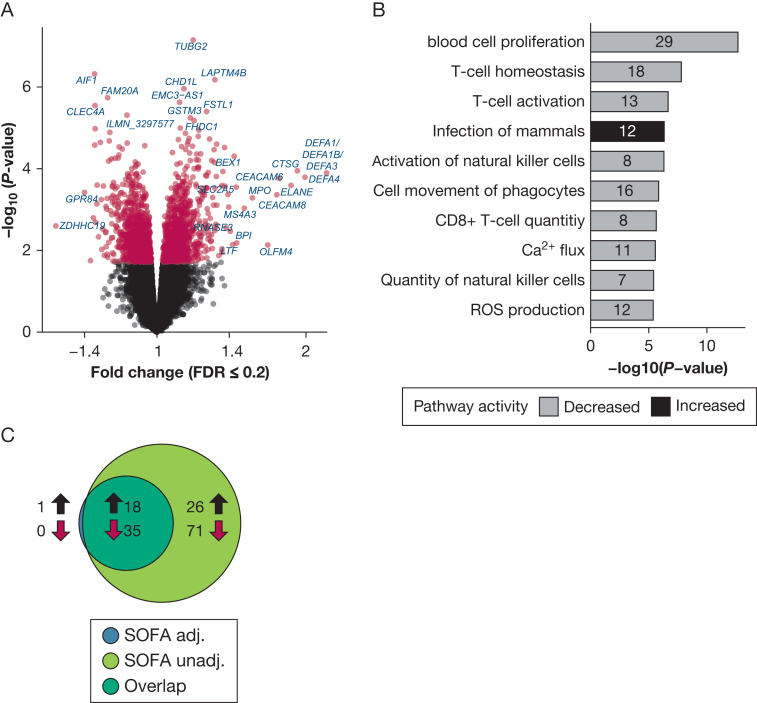
A-C, Whole-blood transcriptome in patients who developed severe community-acquired pneumonia in the discovery cohort. A, Volcano plot of differentially expressed genes. Significantly altered gene expression (FDR ≤ 0.2) is marked in red. B, The 10 top enriched pathways are shown with their activity states. Numbers in bars represent the number of genes observed in the respective pathway. C, Venn-Euler diagram of genes with significantly altered expression in the discovery cohort, the validation cohort, or both, with and without adjustment for SOFA scores. adj. = adjusted; FDR = false discovery rate; ROS = reactive oxygen species; SOFA = Sequential Organ Failure Assessment; unadj. = unadjusted.

We first performed an association analysis comparing patients who did (n = 23) or did not (n = 210) develop severe CAP. About 11% of the genes were differentially expressed between the 2 groups, with most displaying reduced expression (e-Tables 4, 5; Fig 1A). Pathway analysis indicates an impaired adaptive immune response (eg, decreased T-cell activation) and impaired activity of the innate immune response (eg, decreased activity of natural killer cells) (e-Table 6, Fig 1B). Because the effect sizes remained similar after adjustment for the SOFA score, this suggests a SOFA-independent contribution of gene expression to the prediction of a severe disease course (e-Table 7, Fig 1C).

**Figure 2:**
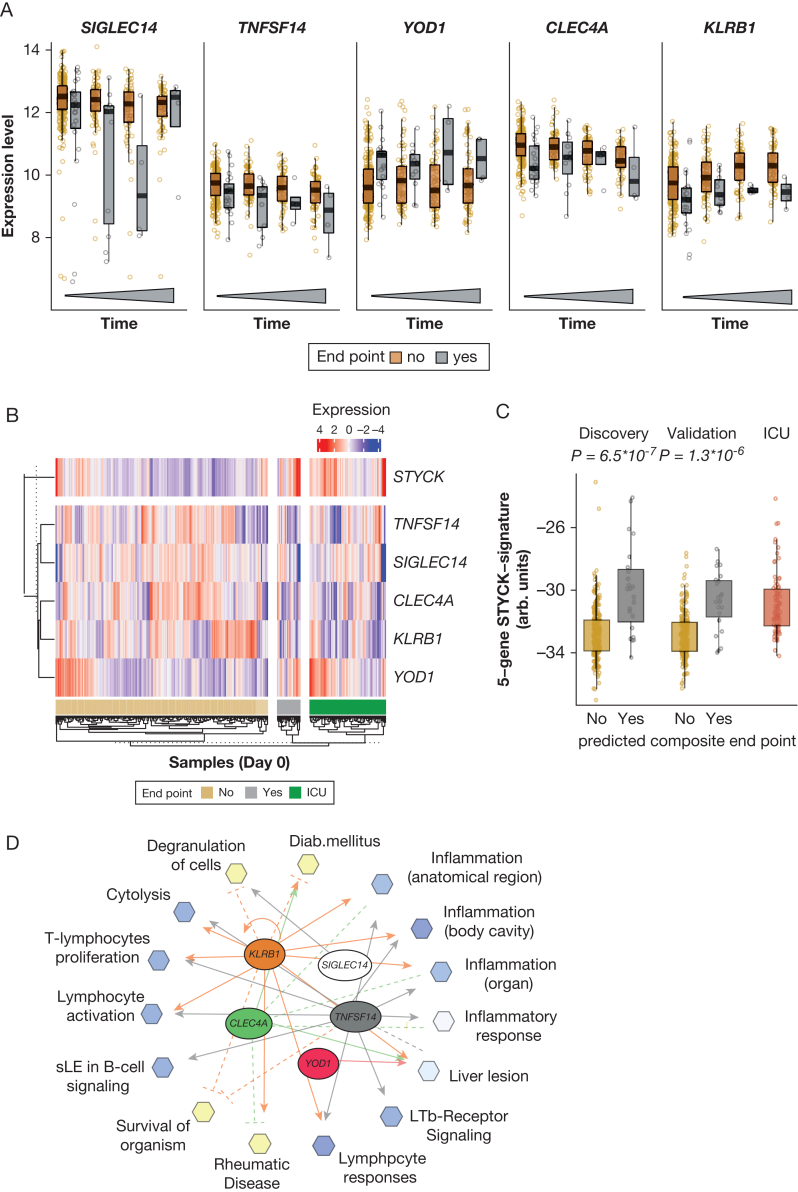
A-D, The 5-gene STYCK signature and its individual genes are differentially expressed in patients with community-acquired pneumonia (CAP) with a severe disease course. A, Boxplot representation of microarray expression levels of the 5 genes of the STYCK signature in patients (gray) or control patients (yellow-brown) in the discovery cohort. Time gradients correspond to the day of study inclusion and the following 3 d at the hospital. B, Heatmap plot of gene expression levels on admission of the 5 genes of the STYCK signature in control patients (left panel), patients with CAP that reached the end point (middle panel), both from the discovery cohort, and patients with CAP directly admitted to ICU. Rows depict genes, and columns depict patients, with the gradient indicating scaled expression levels. Red denotes higher and blue denotes lower expression than the average (shown in white). C, Comparison of the STYCK signature in the discovery and internal validation cohorts and in patients directly admitted to the ICU measured on the day of study inclusion. P values are calculated by the Wilcoxon rank sum test. D, Pathway activation from Ingenuity Pathway Analysis. Yellow and blue hexagons indicate activated and inhibited downstream processes, respectively, as a consequence of the increased expression of YOD1 and decreased expression of the other 4 genes of the STYCK signature in patients with CAP with a severe disease course. Shades of hexagons correspond to the strength of inhibition or activation. arb. = arbitrary; Diab. = diabetes; STYCK = 5-gene signature consisting of *SIGLEC14*, *TNFSF14*, *YOD1*, *CLEC4A*, and *KLRB1*.

Transcriptome data from different days after inclusion were assessed in a time series analysis. Five genes were identified that met our predefined criteria. This 5-gene signature consisting of *SIGLEC14*, *TNFSF14*, *YOD1*, *CLEC4A*, and *KLRB1* (STYCK) included 1 upregulated gene (*YOD1*, involved in the endoplasmic reticulum stress response) and 4 downregulated genes (*SIGLEC14*, *CLEC4A*, and *KLRB1,* all encoding lectin carbohydrate-binding proteins expressed by immune cells, and *TNFSF14*, a member of the tumor necrosis factor [TNF]-ligand family) (e-Tables 8, 9; Figs 2A-C). The 5-gene signature predicted deterioration to severe CAP with an AUC of 0.82 (95% CI, 0.71-0.91; *P* = 6.5 × 10^−7^). This multimarker panel performed better than the individual genes (AUC, 0.61-0.73) (e-Table 10). The STYCK signature was also associated with a severe CAP course in the validation cohort (e-Tables 9, 10; Fig 2C), with a similar AUC (0.81; 95% CI, 0.70-0.91; *P* = 1.3 × 10^−6^), and correlated well with results obtained by next-generation sequencing (e-Figs 3A, 3B). A strong correlation was also observed between the microarray data and TaqMan qPCR measurements (e-Figs 3C, 3D). This supports the robustness of the STYCK signature across different platforms, and transfer to more rapid measurement techniques. Ingenuity Pathway Analysis indicated a predominantly decreased immunologic response consistent with the pathway analysis of all significantly altered genes (e-Table 6, Fig 2D). A sensitivity analysis showed that the effect sizes of the 5 signature genes remained robust after additional adjustment for either preadmission antibiotic use or absolute lymphocyte count (e-Fig 4). These results confirm that the signature’s prognostic performance is independent of these potential confounders.

### STYCK Signature Outperforms Standard Biomarkers/Scores

**Figure 3:**
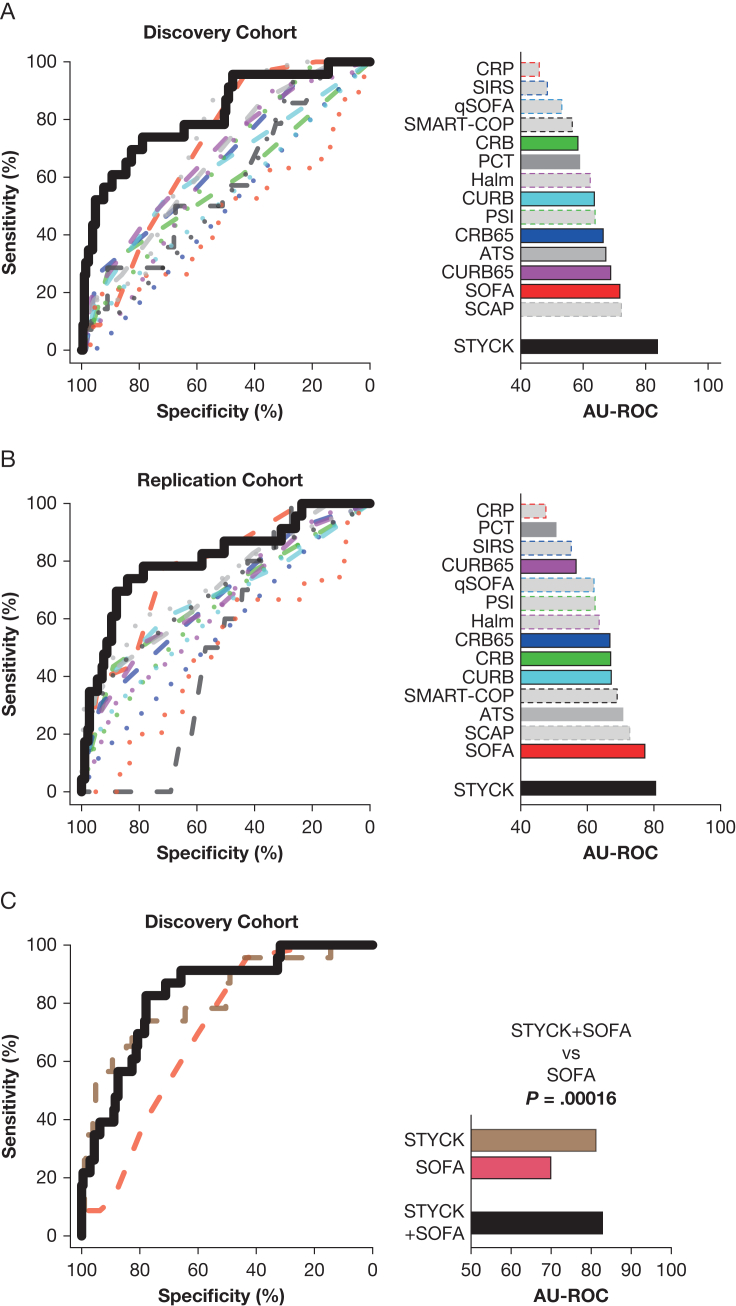

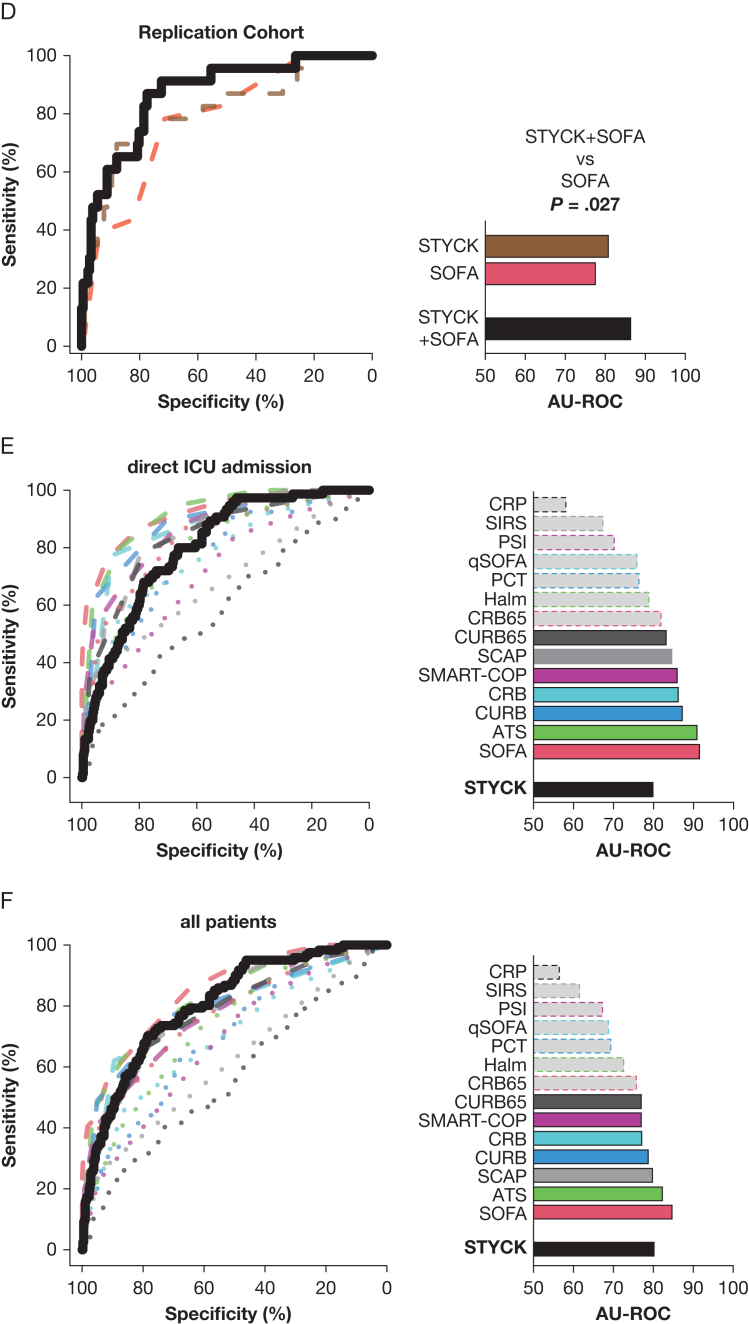

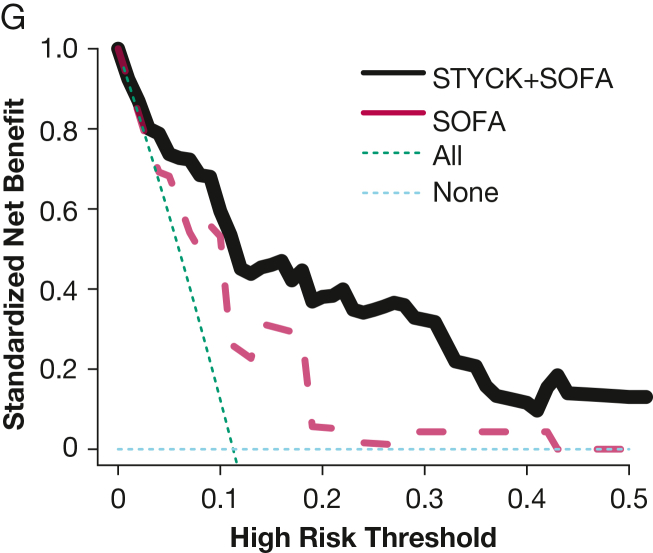
A-F, Prediction performance of the STYCK signature outperforms other clinical scores and serologic markers. A-B, Comparison of the performance of the STYCK signature with indicated clinical scores, CRP, or PCT in (A) the discovery cohort and (B) the validation cohort. C-E, Predictive performance of the combination of the STYCK signature with the SOFA score in (C) the discovery cohort, (D) the validation cohort, and (E) in patients directly admitted to the ICU. F, STYCK performance in all patients analyzed. G, Decision curve analysis: the standardized net benefit at different high-risk thresholds (thresholds for the predicted probability of the outcome at which the clinician decides to treat the patient) of the STYCK + SOFA prediction model (black curve), the SOFA score alone (red curve), treatment for all patients (thin gray line), and treatment for none (x-axis). ATS = American Thoracic Society minor criteria; AU-ROC = Area under receiver operating characteristics; CRB = similar to CURB without blood urea nitrogen; CRB65 = similar to CURB but additionally considering ≥ 65 y of age; CRP = C-reactive protein; CURB = Confusion, Urea, Respiratory rate, BP score; Halm = Halm score; PCT = procalcitonin; PSI = Pneumonia Severity Index (Fine score); qSOFA = quick SOFA; SCAP = severe community-acquired pneumonia score; SIRS = systemic inflammatory response syndrome; SMART-COP = systolic BP, multilobar chest radiographic involvement, low albumin level, high respiratory rate, tachycardia, confusion, poor oxygenation, and low arterial pH score; SOFA = Sequential Organ Failure Assessment score. STYCK = 5-gene signature consisting of *SIGLEC14*, *TNFSF14*, *YOD1*, *CLEC4A*, and *KLRB1*.

The predictive performance of the identified STYCK signature was compared against 2 serologic inflammation markers (C-reactive protein and procalcitonin) and against 12 routinely used clinical.^9^ The STYCK signature had the best predictive performance in both discovery and validation cohorts. The ability to predict severe disease courses was significantly improved when the SOFA score and the STYCK signature were combined (referred to as SOFA+) in contrast with SOFA alone (AUC_discovery_, 0.832 vs 0.703; *P* = .00016 and AUC_validation_, 0.867 vs 0.778; *P* = .027) (Figs 3A-D). This finding was supported by higher scores of the 5-gene STYCK signature in patients with low SOFA scores at study inclusion (e-Fig 3E).

Quantification of predictive performance as the area under the precision-recall curve (AUC-PR) demonstrated better performance of the STYCK signature relative to most of the 14 clinical scores in both cohorts (e-Figs 5A, 5B). We confirmed these results in a sensitivity analysis in which patients with CAP with nonrespiratory indications for ICU admission were excluded (e-Figs 6A-D). Again, in the AUC-PR, the SOFA+ including STYCK signature exhibited superior performance compared with SOFA (AUC-PR_discovery_, 44.3% vs 16.4%, and AUC-PR_validation_, 52.8% vs 33.0%) (e-Figs 5C, 5D, 6E-H). When the STYCK signature was applied to patients either directly admitted to the ICU or to all patients, it did not perform better than the other scores. This supports the potential utility of STYCK in patients not suspected of having severe disease or a high risk of deterioration (e-Figs 7A, 7B; Figs 3E, 3F).

STYCK and the combined STYCK-SOFA predictor showed good discriminatory power for the outcome of 28-day mortality alone and for ICU admission, with slightly higher discriminatory power for 28-day mortality (e-Figs 7C-E). Aiming to evaluate the clinical utility of our prediction model, we next conducted a decision curve analysis.^26^ Here a net benefit of a prediction rule can be determined by weighing true positives (who truly need ICU admission) against false positives (who do not need admission). The decision curve analysis demonstrated a higher standardized net benefit than SOFA score alone for thresholds up to 0.5 (Fig 3G). More specifically, at a 0.15 threshold, the standardized net benefit would be 46.0% (95% CI, 16.1-67.1), indicating the model’s effectiveness is comparable with treating 46.0% of true cases with no false positives. By contrast, the SOFA score alone yielded a 30.7% (95% CI, 9.9-53.2) net benefit at this threshold. We also tested a higher threshold of 0.3, at which STYCK achieved a standardized net benefit of 32.3% (95% CI, 5.7-53.7) compared with 4.3% (95% CI, 0.0-13.6) achieved by the SOFA score alone.

We then considered 2 clinically relevant cutoffs to identify low- and high-risk patients (Table 3). Using the combined STYCK-SOFA score, a negative predictive value of 99% was achieved with a specificity of 51%. In other words, more than one-half of the patients who did not develop the end point were correctly identified with a cutoff of 0.042. By contrast, using the SOFA score alone with the same specificity resulted in a negative predictive value of 96%.

**Table 3:** Modeling of Optimal Cutoffs, Sensitivity, and Specificity of the Clinical Score in Combination or Not With STYCK

Application		Score				
Clinical objective	Parameter	SOFA+STYCK	STYCK	SOFA	PSI	CURB-65
Identifying low-risk patients	Cutoff	0.042^a^	0.036^a^	≤ 2	≤ 2	< 1
	NPV, %	99	96	96	93	95
	Specificity, %	51	36	52	48	23
Identifying high-risk patients	Cutoff	0.398	0.223	≥ 8	5	≥ 4
	PPV	55	41	33	31	50
	Sensitivity	26	52	4	17	22

Performance was assessed at day of inclusion in the validation cohort. CURB65 = confusion, urea, respiratory rate, blood pressure and age ≥ 65; NPV = negative predictive value; PPV = positive predictive value; PSI = Pneumonia Severity Index; SOFA = Sequential Organ Failure Assessment; STYCK = 5-gene signature consisting of *SIGLEC14*, *TNFSF14*, *YOD1*, *CLEC4A*, and *KLRB1*.

a Probability taken from logistic regression modeling in the discovery cohort.

The score was then optimized for positive predictive value (PPV) and sensitivity, with a cutoff probability of 0.398 in the discovery cohort. When applying this cutoff to the validation cohort as a threshold for intensified care, the PPV was 55%, identifying 26% of patients as high risk. Using the SOFA score alone in the validation cohort, performance was reduced, with a PPV of 33% and only 4% of patients identified as high risk. The STYCK-SOFA combination also showed improved performance in identifying both low- and high-risk patients when compared with the Pneumonia Severity Index or CURB-65 scores (Table 3).^27^

### STYCK Signature Predicts Sepsis Mortality

**Figure 4:**
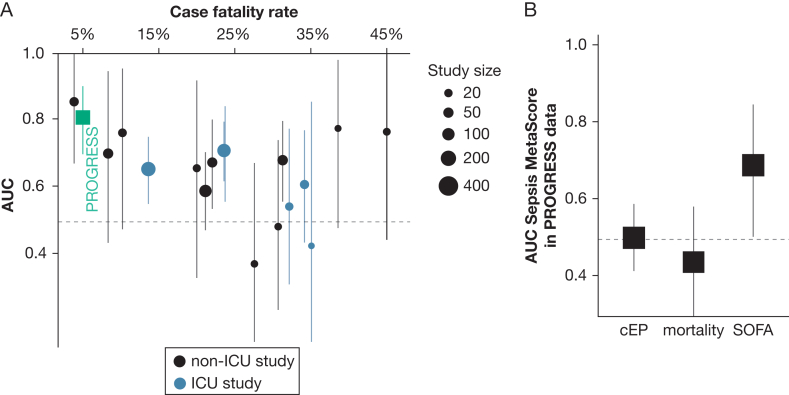
A, B, Five-gene signature consisting of *SIGLEC14*, *TNFSF14*, *YOD1*, *CLEC4A*, and *KLRB1* (STYCK) predicts sepsis mortality. A, STYCK performance in predicting mortality in publicly available data sets with patients with sepsis stratified according to the reported case fatality rate. The green box indicates the PROGRESS validation cohort. Details are provided in e-Table 11. B, Performance of the Sepsis MetaScore^12^ in the combined discovery and validation PROGRESS cohorts for prediction of severe community-acquired pneumonia, 28-d all-cause mortality, and the association with a SOFA score ≥ 7. The horizontal line indicates an AUC of 0.5. Statistical significance was inferred if the 95% CIs did not overlap. AUC = area under the curve; cEP = composite end point; PROGRESS = Progression of Community-Acquired Pneumonia in the Hospital; SOFA = Sequential Organ Failure Assessment.

To obtain external validation of STYCK, the discriminative performance of STYCK signature was analyzed in 17 publicly available sepsis-related data sets predominantly sampled from ICU populations (Fig 4). Sepsis mortality was considerably higher (median, 25%) than in the STYCK validation cohort (median, 5.1%). Nonetheless, significant discriminatory power of the STYCK signature could be confirmed in 6 of the 17 of data sets, notably those including patients at lower mortality risk (e-Table 11, Fig 4A). There was no clear discrimination between mortality risk and AUC (sample size-weighted correlation *r* = −0.36, *P*_bootstrap_ = .16) (Fig 4A). However, when meta-analyzing the 17 external data sets, a significant discrimination was seen (AUC_meta_, 0.66; 95% CI, 0.61-0.70; *P* < .0001). Of note, the Sepsis MetaScore, a previously identified 11 gene-based score originally described for the diagnosis and prognosis of sepsis in trauma patients,^28^ discriminated between more or less severe disease states (using a SOFA cutoff score of 7) at the time of expression measurement in the PROGRESS cohort. However, this Sepsis MetaScore did not predict deterioration to severe CAP in the study cohort in our study (Fig 4B).

## Discussion

Currently used parameters and clinical scores fail to accurately predict significant deterioration of patients hospitalized with CAP, especially at an early point in their hospital admission.^3^ Using an unbiased approach with time series transcriptomics, we identified a new 5 gene-based score (STYCK) that predicts a severe disease course. In patients not immediately admitted to the ICU on clinical triage in the emergency department, it outperformed available clinical prediction tools such as CURB-65^29^ and the Pneumonia Severity Index.^27^ These scores were primarily designed for mortality prediction and not for the dynamic outcome of clinical worsening. Our comparison therefore highlights that these existing tools are not sufficient for this distinct prognostic purpose, underscoring the need for novel predictive biomarkers such as the one we present here. The SOFA+ score (ie, combining with STYCK) improved the performance in predicting a severe CAP course.^9^

As shown in Table 1, a definitive microbiological diagnosis was absent in most of the cohort, which is a common challenge in CAP management. Our study was thus designed to identify a robust, pathogen-agnostic host-response signature that can provide prognostic information even when the causative organism is unknown. The signature’s strong performance in this real-world setting underscores its potential clinical utility. All 5 genes included in the STYCK score regulate adaptive or innate immunity. Three of the genes (*CLEC4A*, *KLRB1*, and *SIGLEC14)* encode lectin carbohydrate-binding proteins that are primarily required to recruit immune cells.^30^ *TNFSF14* is a member of the TNF-ligand family with a potential role in bacterial and viral ARDS. *YOD1* is a deubiquitinating enzyme, also involved in the endoplasmic reticulum stress response with a known function in controlling innate immunity to viral infection.^31^ Expression of *YOD1* correlated highly with 11 other genes that could contribute to the biological effects (e-Table 12). Except for *KLRB1*, none of the transcripts has been previously associated with CAP. Only the combination of the expression of the 5 genes predicted the development of severe CAP and outperformed conventional serum markers and clinical scores in patients that would otherwise not have been identified based on clinical assessment.

The end point in the cohort was associated with downregulated transcription of genes encoding lectins and *TNFSF14* and upregulated expression of a gene involved in endoplasmic reticulum-associated protein degradation in the activation of a viral host response.^31^ The genes could constitute genetic risk factors, as has been suggested in smaller cohorts (eg, for genetic variants of interferon-λ-3 or TNF for COVID-19 severity,^32^^,^^33^ for TNF in the development of pulmonary sepsis^34^).

STYCK also performed well in sepsis-related cohorts, preferentially derived from non-ICU studies and those with low case fatality rates.^16^^,^^20^^,^^35^, ^36^, ^37^, ^38^ As severe disease courses are rare in these settings, comparatively unexpected, and their onset easily overlooked, prediction reflects an important unmet medical need.

In external sepsis data sets, the performance of the STYCK signature worsened when mortality increased. A potentially relevant difference to the cohort is the inclusion of patients not deemed to be sick enough for ICU admission. STYCK was established and trained on a low-mortality cohort, and this may explain why it performs best on cohorts with similar mortality rates. The varying performance of the STYCK signature across different mortality rates highlights the need for further validation and calibration of the signature in diverse patient populations. It is possible that biological processes captured by the STYCK signature are more relevant in the early stages of disease progression, and other factors may become more important as the disease severity increases.

With increasing pressures on ICU capacity, hospital-at-home programs are currently being developed for a broad range of patients including those receiving acute-level care (eg, IV antibiotics, monitoring, skilled nursing care). This approach could lower complications (eg, delirium, nosocomial infection) and reduce costs compared with prolonged inpatient stays.^39^ The key requirement is considered careful patient selection and the ability to monitor, escalate, and rapidly transfer patients to hospital if their condition worsens.^40^ CAP, with its high incidence and broad range of severities from mild to ARDS, would be a prime condition suitable for such programs in particular if reliable predictions regarding incipient deterioration, as we propose based on our STYCK transcriptomic signature, become available.

This study has several limitations. We included all-cause mortality, potentially affecting specificity of the identified STYCK signature. Most of the included patients with CAP had a nonsevere disease course (ie, only a small number of participants reached the predefined end point). We assumed missing values to be within normal ranges. This could have led to a biased estimation of clinical score performance. Patients were admitted with variable symptoms, and this could affect both type and extent of gene transcription. This was not accounted for in the study population. Although we sought to obtain external validation of our STYCK signature, appropriate cohorts with comparable disease states and end points are currently unavailable. A further limitation is the real-world heterogeneity of the patient cohort. Day 0 sampling occurred at a median of about 21 hours after admission, and patients presented with varying durations of symptoms before hospitalization. Although the signature proved robust in this setting, its performance was not stratified by these specific time variables. Furthermore, because the signature was evaluated in hospitalized patients, its utility for prognosis in an outpatient or emergency department setting remains to be determined.

## Interpretation

We identified that the STYCK signature taken soon after hospital admission predicts a severe CAP disease course. It outperformed conventional markers and scores in predicting ICU admission or death in patients who were not immediately admitted to intensive care. The predictive performance in other available data sets suggests that the signature could be useful beyond hospitalized CAP; however, this needs confirmation by subsequent studies. Although our study provides strong evidence from a large prospective cohort and extensive external validation, future research should aim to prospectively test the STYCK signature’s clinical utility and to evaluate whether early risk stratification using the signature can effectively guide clinical decisions and improve patient outcomes.

## Funding/Support

The PROGRESS study is funded by the 10.13039/501100002347German Federal Ministry of Education and Research (BMBF) [Grants 01KI07111 (M. B., M. K.), 01KI07114 (N. S., M. W., P. C.), 01KI07113, 01KI1010I (H. K., P. A., M. L., M. S.)]. Additional funding is provided by the 10.13039/501100010564German Center for Lung Research (DZL) [Grants 82DZLJ19A1, 82DZLJ19A2, 82DZLJ19B1, 82DZLJ19B2, 82DZLJ19C1, 82DZLJ19C2]. H. K., P. A., and M. S. were also supported by CAPSyS [Grant 01ZX1304A] and SYMPATH-1 and SYMPATH-2 [Grants 01ZX1906B, 01ZX2206B], and H. K. and P. A. were also supported by the German Federal Ministry of Education and Research (BMBF) within the project "Center for Scalable Data Analytics and Artificial Intelligence (ScaDS.AI) Dresden/Leipzig” [BMBF Grant 01IS18026B]. S. W. and M. B. were also funded by the German Ministry of Education and Research (BMBF) [Grant 01 EO 1502] via the Jena Center of Sepsis Control and Care. The RNA sequencing study was funded by the Fraunhofer Zukunftsstiftung (Future Foundation). N. S. received funding for research from the Deutsche Forschungsgemeinschaft (DFG) and SFB-TR84 subproject C09. M. W. received funding for research from the Deutsche Forschungsgemeinschaft (DFG, German Research Foundation), SFB-TR84 (114933180) subprojects C06 and C09, SFB 1449 (431232613) subproject B2, and from the German Ministry of Education and Research (BMBF) in the framework of CAPSyS [Grants 01ZX1604B, 01ZX1304B], SYMPATH [Grant 01ZX1906A], PROVID [Grant 01KI20160A], Phage4Cure [Grant 16GW0141], and MAPVAP [Grant 01KI2124]. G. N. received funding from the DFG, SFB 1449 (Grant 431232613], and BMBF in the framework of CAPSyS [Grants 01ZX1604B, 01ZX1304B], SYMPATH [Grant 01ZX1906A], and MAPVAP [Grant 01KI2124]. M. B. is supported by the Deutsche Forschungsgemeinschaft (German Research Foundation) under Germanys Excellence Strategy [Grant EXC 2051]. S. W. is currently funded by the Deutsche Forschungsgemeinschaft (DFG) [Project WE 4971/6-1], the Excellence Cluster Balance of the Microverse (EXC 2051) [Grant 390713860], the Federal Ministry of Education and Research (BMBF) [Project 01EN2001], and the Horizon 2020 Framework program [Grant 847422].

## Financial/Nonfinancial Disclosures

The authors have reported to *CHEST Pulmonary* the following: H. K., P. A., M. K., P. C., M. L., N. S., M. S., and M. B. filed a patent including the STYCK signature (European Patent Office File No. EP23205776). None declared (S. W., M. W., B. P. S., K. K., M. Rade, F. H., C. Bertram, K. R., D. L., C. Blumert, S. J., K. S., G. N., M. Rosolowski).

## Supplementary Data

e-Online Data

## References

[1] Kolditz M., Tesch F., Mocke L., Hoffken G., Ewig S., Schmitt J. (2016). Burden and risk factors of ambulatory or hospitalized CAP: a population based cohort study. Respir Med.

[2] Jain S., Self W.H., Wunderink R.G. (2015). Community-acquired pneumonia requiring hospitalization among U.S. adults. N Engl J Med.

[3] Gearhart A.M., Furmanek S., English C., Ramirez J., Cavallazzi R. (2019). Predicting the need for ICU admission in community-acquired pneumonia. Respir Med.

[4] Renaud B., Brun-Buisson C., Santin A. (2012). Outcomes of early, late, and no admission to the intensive care unit for patients hospitalized with community-acquired pneumonia. Acad Emerg Med.

[5] Restrepo M.I., Mortensen E.M., Rello J., Brody J., Anzueto A. (2010). Late admission to the ICU in patients with community-acquired pneumonia is associated with higher mortality. Chest.

[6] Dela Cruz C.S., Wunderink R.G., Christiani D.C. (2018). Future research directions in pneumonia. NHLBI Working Group Report. Am J Respir Crit Care Med.

[7] Sibila O., Restrepo M.I. (2019). Biomarkers in community-acquired pneumonia: still searching for the one. Eur Respir J.

[8] Ramirez P., Ferrer M., Marti V. (2011). Inflammatory biomarkers and prediction for intensive care unit admission in severe community-acquired pneumonia. Crit Care Med.

[9] Ahnert P., Creutz P., Horn K. (2019). Sequential organ failure assessment score is an excellent operationalization of disease severity of adult patients with hospitalized community acquired pneumonia - results from the prospective observational PROGRESS study. Crit Care.

[10] Scicluna B.P., Klein Klouwenberg P.M.C., van Vught L.A. (2015). A molecular biomarker to diagnose community-acquired pneumonia on intensive care unit admission. Am J Respir Crit Care Med.

[11] Sweeney T.E., Perumal T.M., Henao R. (2018). A community approach to mortality prediction in sepsis via gene expression analysis. Nat Commun.

[12] Tsalik E.L., Langley R.J., Dinwiddie D.L. (2014). An integrated transcriptome and expressed variant analysis of sepsis survival and death. Genome Med.

[13] Wong H.R., Cvijanovich N.Z., Anas N. (2015). Developing a clinically feasible personalized medicine approach to pediatric septic shock. Am J Respir Crit Care Med.

[14] Wong H.R., Shanley T.P., Sakthivel B. (2007). Genome-level expression profiles in pediatric septic shock indicate a role for altered zinc homeostasis in poor outcome. Physiol Genomics.

[15] van Vught L.A., Scicluna B.P., Wiewel M.A. (2016). Comparative analysis of the host response to community-acquired and hospital-acquired pneumonia in critically ill patients. Am J Respir Crit Care Med.

[16] Sweeney T.E., Shidham A., Wong H.R., Khatri P. (2015). A comprehensive time-course-based multicohort analysis of sepsis and sterile inflammation reveals a robust diagnostic gene set. Sci Transl Med.

[17] Baghela A., Pena O.M., Lee A.H. (2022). Predicting sepsis severity at first clinical presentation: the role of endotypes and mechanistic signatures. EBioMedicine.

[18] Bauer M., Giamarellos-Bourboulis E.J., Kortgen A. (2016). A transcriptomic biomarker to quantify systemic inflammation in sepsis - a prospective multicenter phase II diagnostic study. EBioMedicine.

[19] Almansa R., Heredia-Rodriguez M., Gomez-Sanchez E. (2015). Transcriptomic correlates of organ failure extent in sepsis. J Infect.

[20] Ahnert P., Creutz P., Scholz M. (2016). PROGRESS - prospective observational study on hospitalized community acquired pneumonia. BMC Pulm Med.

[21] Sweeney T.E., Wong H.R., Khatri P. (2016). Robust classification of bacterial and viral infections via integrated host gene expression diagnostics. Sci Transl Med.

[22] European Medicines Agency Guideline for good clinical practice E6(R2). https://www.ema.europa.eu/en/documents/scientific-guideline/ich-e-6-r2-guideline-good-clinical-practice-step-5_en.pdf.

[23] World Medical Association WMA Declaration of Helsinki – ethical principles for medical research involving human participants. https://www.wma.net/policies-post/wma-declaration-of-helsinki-ethical-principles-for-medical-research-involving-human-subjects/.

[24] Newman A.M., Liu C.L., Green M.R. (2015). Robust enumeration of cell subsets from tissue expression profiles. Nat Methods.

[25] Krämer A., Green J., Pollard J., Tugendreich S. (2014). Causal analysis approaches in Ingenuity Pathway Analysis. Bioinformatics.

[26] Vickers A.J., Elkin E.B. (2006). Decision curve analysis: a novel method for evaluating prediction models. Med Decis Making.

[27] Aujesky D., Auble T.E., Yealy D.M. (2005). Prospective comparison of three validated prediction rules for prognosis in community-acquired pneumonia. Am J Med.

[28] Sweeney T.E., Wong H.R. (2016). Risk stratification and prognosis in sepsis: What have we learned from microarrays?. Clin Chest Med.

[29] Lim W.S., van der Eerden M.M., Laing R. (2003). Defining community acquired pneumonia severity on presentation to hospital: an international derivation and validation study. Thorax.

[30] Chiffoleau E. (2018). C-type lectin-like receptors as emerging orchestrators of sterile inflammation represent potential therapeutic targets. Front Immunol.

[31] Liu C., Huang S., Wang X. (2019). The Otubain YOD1 suppresses aggregation and activation of the signaling adaptor MAVS through Lys63-linked deubiquitination. J Immunol.

[32] Vakil M.K., Mansoori Y., Al-Awsi G.R.L. (2022). Individual genetic variability mainly of Proinflammatory cytokines, cytokine receptors, and toll-like receptors dictates pathophysiology of COVID-19 disease. J Med Virol.

[33] Agwa S.H.A., Kamel M.M., Elghazaly H. (2021). Association between interferon-lambda-3 rs12979860, TLL1 rs17047200 and DDR1 rs4618569 variant polymorphisms with the course and outcome of SARS-CoV-2 patients. Genes (Basel).

[34] Feng B., Mao Z.R., Pang K., Zhang S.L., Li L. (2015). Association of tumor necrosis factor alpha -308G/A and interleukin-6 -174G/C gene polymorphism with pneumonia-induced sepsis. J Crit Care.

[35] Parnell G.P., Tang B.M., Nalos M. (2013). Identifying key regulatory genes in the whole blood of septic patients to monitor underlying immune dysfunctions. Shock.

[36] Ahn S.H., Tsalik E.L., Cyr D.D. (2013). Gene expression-based classifiers identify Staphylococcus aureus infection in mice and humans. PLoS One.

[37] Kangelaris K.N., Prakash A., Liu K.D. (2015). Increased expression of neutrophil-related genes in patients with early sepsis-induced ARDS. Am J Physiol Lung Cell Mol Physiol.

[38] Pankla R., Buddhisa S., Berry M. (2009). Genomic transcriptional profiling identifies a candidate blood biomarker signature for the diagnosis of septicemic melioidosis. Genome Biol.

[39] Rangarajan S., Jenq G., Web exclusive (2022). Annals for hospitalists inpatient notes - the future of hospital-at-home care. Ann Intern Med.

[40] Paulson M.R., Torres-Guzman R.A., Matcha G.V. (2023). Treatment of a high healthcare utilizer with sepsis in a virtual hybrid hospital-at-home program. Clin Case Rep.

