# Supplementary material for: Community-Acquired Pneumonia: Disease Course Prediction With a 5-Gene Signature
Source: CHEST Pulm. 2026 Mar 10;4(2):100256. doi: 10.1016/j.chpulm.2026.100256 (PMC13418022; doi:10.1016/j.chpulm.2026.100256)
Supplement: e-Online Data [file mmc1.docx]

**Online Data Supplement:**

**Community Acquired Pneumonia - Disease Course Prediction with a 5-Gene Signature**

Holger Kirsten^1^*, Sebastian Weis^2,3,4^*, Peter Ahnert^1^, Martin Witzenrath^5,6^, Brendon P. Scicluna^7,8^, Knut Krohn^9^, Michael Rade^10^, Friedemann Horn^10^ Catharina Bertram^10^, Kristin Reiche^10^, Dennis Löffler^10^, Conny Blumert^10^, Stefan Jenner^11^, Kai Sohn^11^, Geraldine Nouailles^5^, Michael Kiehntopf^12,13^, Petra Creutz^5^, Maciej Rosolowski^1^, Markus Loeffler^1^, Norbert Suttorp^5,6^, PROGRESS Study Group, Markus Scholz^1+^, Michael Bauer^2+^

**PROGRESS Study Group:**

BG Universitätsklinikum Bergmannsheil GmbH, Klinik für Pneumologie, Allergologie und Schlafmedizin: Barabara Hauptmeier, Deborah Wehde; Charité - Universitätsmedizin Berlin, Medizinische Klinik mit Schwerpunkt. Infektiologie und Pneumologie: Michael Benzke, Ulrike Föllmer, Carmen Garcia, Andreas Hocke, Charité ICU-Teams, Charlotte Keller, Agata Mikolajewska, Michaela Niebank, Mirja Ramke; Charité - Universitätsmedizin Berlin, Klinik für Anästhesiologie und Intensivtherapie: Stefan Angermair; Christliches Krankenhaus Quakenbrück e. V., Abteilung Pneumologie, Allergologie, Schlafmedizin: Oleg Dmitriev, Hermann Druckmiller; Galina Schott; Diakoniekrankenhaus Rotenburg (Wümme) gGmbH, Zentrum für Pneumologie: Tom Schaberg, Iris Hering; Evangelische Kliniken Bonn, Innere Medizin II: Martin Buchenroth, Oliver Kanwar; Evangelisches Krankenhaus Kalk gGmbH, Innere Medizin / Pneumologie: Lena Kappauf; Gemeinschaftskrankenhaus Havelhöhe, Kardio-Pneumologie: Christian Grah; Kliniken des Main-Taunus-Kreises, Klinik für Pneumologie und Allgemeine Innere Medizin: Carola Hobler, Simone Hamberger, Thomas Müller; Klinikum Dortmund gGmbH, Medizinische Klinik (Pneumologie / Infektiologie): Frederik Hempel, Kalina Popkirova, Bernhard Schaaf, Markus Unnewehr; Klinikum St. Georg gGmbH, Klinik für Infektions-/Tropenmedizin und Nephrologie: Bernhard Ruf, Nils Keller; Klinikum Würzburg MissioKlinik gGmbH, Medizinische Klinik mit Schwerpunkt Pneumologie und Beatmungsmedizin: Matthias Held, Barbara Schröder; Klinken Heidenheim, Medizinische Klinik II: Brigitte Mayer; Krankenhaus Angermünde, Klinik für Innere Medizin / Pneumologie: Christoph Arntzen, Jan Pluta; Krankenhaus Bad Arolsen GmbH, Innere Medizin (Herz-, Kreislauf- und Lungendiagnostik): Walter Knüppel; Krankenhaus München-Neuperlach, Klinik für Kardiologie, Pneumologie und Internistische Intensivmedizin: Phillipp Mayer, Christine Krollmann, Thomas Spinner; LKH-Universität Klinikum Graz, UKIM Pulmologie: Holger Flick, Gudrun Wakonigg; Lungenklinik Ballenstedt/Harz gGmbH, Evangelisches Fachkrankenhaus für Lungenkrankheiten: Barbara Wagener; Mathias-Spital Rheine, Klinik für Pneumologie und Thoraxonkologie, Medizinische Klinik V: Ayhan Berber, Nicolas Dickgreber; Medizinische Hochschule Hannover, Klinik für Pneumologie: Julia Freise, Jessica Rademacher; St. Vincenz und Elisabeth Hospital, Innere Medizin: Andreas Liebrich; St. Vincentius-Kliniken gAG, Med. Klinik IV / Pneumologie: Michael Simpfendörfer; Städt. Klinikum Dessau, Innere Medizin: Judith Pannier, Mathias Plauth, Marianne Schelle; Städtisches Klinikum München GmbH, Klinik für Akut- und Internistische Intensivmedizin: Claus Peckelsen, Patrick Schmidt-Schridde; Universität Leipzig, Innere Medizin, Neurologie und Dermatologie, Pneumologie: Lea Deterding, Eva Koch, Hubert Wirtz; Universitätsklinikum Carl Gustav Carus, TU Dresden, Medizinische Klinik 1 - Abteilung Pneumologie: Martin Kolditz; Universitätsklinikum des Saarlandes, Innere Medizin V: Robert Bals, Christian Lensch; Universitätsklinikum Hamburg Eppendorf, Onkologisches Zentrum, Pneumologische Studienzentrale: Tim Oqueka; Universitätsklinikum Jena, Klinik für Anästhesiologie und Intensivtherapie: Katrin Ludewig, Frank Bloos, Daniel Thomas-Rüddel, Universitätsklinikum Jena, Institut für Infektionsmedizin und Krankenhaushygiene: Anne Moeser, Mathias Pletz; Universitätsklinikum Münster, Innere Medizin, Intensivmedizin: Felix Rosenow, Lena-Maria Makowski; Universitätsklinikum Schleswig-Holstein, Medizin 1: Lorenz Balke; Universitätsklinikum Schleswig-Holstein, Med. Klinik III (Pneumologie): Jan Rupp; Universitätsklinikum Ulm, Studienzentrale Innere II, Pneumologie: Cornelia Kropf-Sanchen, Stefan Rüdiger; Universitätsmedizin Mannheim, Studienkoordinierungszentrum, 1. Medizinische Klinik: Ursula Hoffmann, Tetyana Shchetynska-Marinova; Vivantes Humboldt-Klinikum, Kardiologie und kons. Intensivmedizin: Josefa Lehmke; Vivantes Klinikum Spandau, Kard., Pneum. und kons. Intensivmedizin: Sven Gläser, Henning Kahnert, Markus Niesen; Vivantes Netzwerk für Gesundheit GmbH Vivantes Klinikum Neukölln, Klinik für Innere Medizin - Pneumologie & Infektiologie – Thoraxzentrum: Achim Lies, Wulf Pankow, Dorina Thiemig

**SUPPLEMENTARY METHODS**

**Study design and participants**

The Study of Progression of Community Acquired Pneumonia in the Hospital (PROGRESS) was a prospective multicenter observational cohort study that was recruiting CAP patients requiring hospitalization in Germany and Austria (ClinicalTrials.gov: [NCT02782013](https://www.clinicaltrials.gov/ct2/show/NCT02782013)) from 09/2009 to 04/2017 [1]. The overall aim of the PROGRESS study was to identify clinical and molecular genetic factors determining or predicting severe disease courses of CAP patients. Patients older than 18 years with a diagnosis of CAP were considered for inclusion. CAP was defined as *i.*) pulmonary infiltrate detected by chest X-ray, and *ii.*) at least two of the following symptoms: fever, cough, purulent sputum, shortness of breath/need for respiratory support, or crackling /rales on auscultation, dullness to percussion, or bronchial breathing. Exclusion criteria were patients *i.*) admitted to hospital within the previous 28 days, *ii*.) with immunosuppression and *iii.*) with therapy limitations. A detailed list has been published [2].

The PROGRESS protocol allowed enrollment within 48 hours of hospital admission with confirmed CAP, while an extension of the protocol, called PROGRESS-CAPSyS, allowed patients to be enrolled within seven days of admission. In our data set, 55% of the participants were enrolled within 24 hours, and 99.5% were enrolled within 48 hours after hospital admission.

The disease course and the therapy of patients were closely monitored by daily checks and long-term follow-up. Comorbidities were assessed using the modified Charlson score [3].

Whole blood samples for gene expression analysis were sampled between day 0 (day of enrollment, referred to as, study inclusion and day 4. We analyzed these time series of gene-expressions to select features for our signature, while signature performance was tested on the cross-sectional data collected at baseline.

Further details on sample and data collection were described in the published study protocol [2]. For the presented PROGRESS sub-study, we included participants that were recruited between 09/2009 and 04/2017. The contribution of each of the 60 study sites is shown in *e-Table.1*. We required patients with available gene expression measurements and excluded patients not fulfilling our criteria for qualified ICU admission as specified below (see also *e-Fig.1* for a study flow chart). In accordance with the methodology outlined by Fine *et al.* [4] and subsequently applied by Ahnert *et al.,* [2] missing parameters in the clinical score calculation were replaced by uncritical values and missing parameters in the SOFA score calculation were implemented by the "last observation carried forward" technique.

The study protocol was approved by the ethics committee of the University of Jena (2403–10/08) and by locally responsible ethics committees for each study site. The study was conducted in accordance with Good Clinical Practice guidelines [5] and the provisions of the Declaration of Helsinki [6]. Written informed consent was obtained from the patients or their legal representatives. All collected biomaterials were stored in a central biobank, and clinical data were stored in a central database.

**Generation of the discovery cohort and validation cohort**

To prevent bias in the development and testing of the gene signature, the final study population of 455 patients was randomly separated into a discovery cohort (n=240) and a validation cohort (n=215) using a robust, programmatic approach designed to ensure cohort comparability.

The randomization procedure was implemented via a custom R script and followed these specific steps:

**1. Stratification:** The initial randomization was stratified for two critical variables: (i) the composite endpoint status (worsening/death) and (ii) the presence of missing data for covariates required in the regression models. This step ensured these fundamental characteristics were distributed proportionally between the two cohorts.

**2. Iterative balancing:** A simple random split was deemed insufficient to guarantee balance across all key clinical characteristics. Therefore, we implemented an iterative probabilistic sampling loop. This algorithm performed a random split of the cohort and then immediately conducted a series of statistical comparisons between the newly created discovery and validation sets.

**3. Balance assessment:** The algorithm automatically compared the distributions of over 30 pre-specified clinical, demographic, and laboratory variables. This comprehensive list included:

- **Demographics and Vitals:** Age, sex, body mass index (BMI), smoking status, and smoking pack-years.

- **Outcomes:** Need for mechanical ventilation, ICU admission, subsequent mortality, and achievement of the composite endpoint.

- **Severity scores:** The CURB-65 and CRB-65 scores, the baseline SOFA score, the worst-observed future SOFA score, and all individual SOFA sub-scores (pulmonary, cardiovascular, coagulation, renal, liver, and CNS).

- **Laboratory values:** C-reactive protein (CRP), procalcitonin (PCT), total leukocyte count, and differential blood counts including percentages of lymphocytes, neutrophils, monocytes, eosinophils, and basophils.

- **Comorbidities:** The overall comorbidity score.

- **Study-specific variables:** Enrollment in the CAPSyS protocol, the timepoint of measurement, and the number of timepoints where patients were measured.

**4. Acceptance Criteria:** If any of these comparisons yielded a nominal p-value of <0.1 regarding the first measure of a patient’s time-series or p-value of <0.05 regarding the last measure of a patient’s time-series, the split was automatically rejected. The algorithm then returned to step 2 and generated a new random split. This process was repeated until a randomization was achieved where none of the key baseline characteristics differed significantly between the discovery and validation cohorts and improvement of balancing did not improve anymore.

**RNA isolation and globin transcript depletion**

For transcriptome analysis, whole venous blood was collected into PAXgene blood RNA tubes (Qiagen, Hilden, Germany) and stored at -80 °C until further processing. mRNA was isolated with a PAXgene miRNA kit (Qiagen). In brief, frozen whole blood was thawed and equilibrated at room temperature for 2 h. The samples were centrifuged at 4,000g, the supernatant was decanted, and the pellets were resuspended in 4 mL of water. Further purification was carried out according to the manufacturer's instructions using a QIAcube. After two steps of DNase digestion (TURBO DNA-free Kit, Ambion) and sample concentration (RNA Clean & Concentrator-5 Kit, Zymo Research), the extracted RNA was quantified using a Qubit RNA Kit and a DeNovix instrument (Biozym). or Nanodrop 2000c (Thermo Scientific). The quality of RNA was assessed with a Bioanalyzer 2100 instrument (Agilent Technologies, Santa Clara, California, USA) with the RNA 6000 Nano Kit. Finally, for samples later used in RNA sequencing, globin mRNA was removed (max. RNA input: 1 µg per sample) using a GLOBINclear™-Human Kit (Ambion), and the concentration and quality of the RNA were determined as described above.

**Array-based gene expression quantification**

Purified RNA was dissolved at a concentration of 50–300 ng/μL prior to probe synthesis. Samples were hybridized to Illumina HT-12 v4 Expression BeadChips (Illumina, San Diego, CA, USA), and hybridization was measured using an Illumina HiScan according to the manufacturer’s instructions. Raw data for all 47,231 gene-expression probes were extracted by Illumina GenomeStudio without additional background correction. The data were further processed using R (version 3.4.3). Expression values were log2-transformed and quantile-normalized to allow parametric analysis [7]. Batch effects of BeadChip expression were corrected using an empirical Bayes method [8]. During preprocessing, gene-expression probes detected by Illumina GenomeStudio as being expressed in less than 5% of the samples were excluded, as were probes still found to be significantly associated with processing batches after Bonferroni correction. These filters resulted in 26,601 valid gene-expression probes corresponding to 16,329 unique genes included in this analysis. Three criteria were used to remove samples of low quality [8]. First, the number of detected gene-expression probes of a sample was required to be within ± 4 interquartile ranges (IQRs) from the median. Second, the Mahalanobis distance of several quality characteristics of each sample was used (“signal of biotin-control probes, signal of low-concentration control probes, signal of medium-concentration control probes, signal of mismatch control probes, signal of negative control probes and signal of perfect-match control probes”) [9]. This had to be within median + 4 x IQR. Third, Euclidean distances of expression values had [7] to be within 5 x IQR from the median.

**Sequencing-based gene expression quantification**

*Library generation and sequencing*

Library preparation and sequencing was performed using 100 ng of globin-depleted RNA using the ScriptSeqTM Complete Gold Kit (Human/Mouse/Rat according to the manufacturer's instructions, which also included ribosomal RNA depletion. AmpureXP Beads (Beckman Coulter) were used for all purification steps. High-throughput sequencing of libraries was performed with HiSeq2500 sequencing (Illumina, San Diego, CA, USA), with an average sequencing depth of 100 million clusters per sample and 2x 100b paired-end reads

*RNA sequencing gene expression quantification and expression quality filtering*

To facilitate the multistep analysis of the RNA sequencing datasets, we applied the workflow manager UAP [10].

*Primary and secondary RNA sequencing data analysis*

Demultiplexing of Illumina raw files was performed with Illumina bcl2fastq software v2.19 (https://emea.support.illumina.com/sequencing/sequencing_software/bcl2fastq-conversion-software.html). Paired-end FASTQ reads were trimmed and filtered using AdaptorRemoval v2.2.1 [11] with additional parameters to trim ambiguous bases (N) at the 5'/3' termini (--trimns), remove low-quality bases (--trimqualities, --minquality 20) and keep reads with a minimum read length of 30 bp (--minlength 30). Transcript abundancy estimation of each sample was conducted using Kallisto v0.44.0 [12] by specifying a forward-stranded library. The human transcriptome FASTA file was downloaded from GENCODE (release 28 GRCh38.p12) and used to create a Kallisto index. Gene-level quantifications were generated from the Kallisto-estimated counts per transcript using tximport v1.8.0 with default parameters [13].

*RNA sequencing quality control*

For each sample, a subsample of 1 million trimmed paired-end reads was randomly chosen by fastq-sample v0.8 (https://homes.cs.washington.edu/~dcjones/fastq-tools/) using default parameters. Sample quality control was determined using FastQC v0.11.5 (https://www.bioinformatics.babraham.ac.uk/projects/fastqc/) to assess base call accuracy and Preseq v2.0.2 [14] to evaluate library complexity. These subsamples were aligned to the human reference genome GRCh38/hg38 using HISAT2 v2.10 [15]. Duplication metrics were collected using the Picard tools v2.3.0 (http://broadinstitute.github.io/picard/) function MarkDuplicates using BAM files generated by HISAT2. Picard’s CollectRnaSeqMetrics function was used to determine mapping percentages on intergenic, intronic, coding and UTR regions as well as gene body coverage. RSeQC v2.6.4 [16] was applied to determine the read GC content, junction saturation, read pair inner distance, and strandness of reads. Aggregated data visualization for the secondary analysis and quality control was performed using the MultiQC framework [17]. FastQ Screen v0.11.1a [18] in conjunction with bowtie2 [19] was applied to assess RNA library composition. In addition, using an iterative approach, we calculated the fraction of reads mapped against human rRNA transcripts to assess rRNA depletion efficacy during RNA library preparation.

For principal component analysis (PCA) and NGS correlation analysis, read counts were normalized using a variance-stabilizing transformation implemented in DESeq2 v1.20.0 [20]; the option “blind = TRUE” was applied to compare samples in an unbiased manner. PCA of samples was performed based on the 5,000 most variable genes.

All next-generation sequencing (NGS) samples were included in correlation analysis, as its aim was to identify transcripts that correlate robustly when comparing data from expression chips and NGS, even in the presence of NGS-quality issues.

*Gene expression and pathway association analysis*

Only expression data measured before reaching the endpoint were analyzed. Gene expression association analysis was performed in the discovery cohort first, applying a mixed effects model as implemented in the R package limma 3.38.3 [21]. Repeated measurements of gene expression were considered as random intercepts and we adjusted for covariates age, sex, BMI, smoking within the last 12 months, comorbidities, SOFA score, and relative proportion of neutrophils and lymphocytes. Missing percentages of neutrophils and lymphocytes among leukocytes were inferred using CIBERSORT [22]. The false discovery rate (FDR) and proportion of null values of all tested hypotheses (Eta 1) were calculated based on empirical null modeling and Grenander-density approaches, as implemented in the R package fdrtool1.2.15 [23]. For pathway enrichment and activation analysis, we used Ingenuity Pathway Analysis [24]. When analyzing the correlation of effect sizes from two different association analyses, we quantified the overall similarity R^2^ as the explained variance in a linear regression model.

**qRT-PCR**

Each sample was determined in triplicates. 130 ng of RNA were reverse-transcribed into 10 µL cDNA using the SuperScript™ VILO™ Master Mix (Invitrogen). For quantitative real-time PCR (qRT-PCR), 2 µL cDNA template (1:10 diluted) was added to the TaqMan^TM^ Fast Advanced Master-Mix (Applied Biosystems) and the respective TaqMan^TM^ Assay (Thermo Fisher Scientific) according to the manufacturer’s protocol. qRT-PCR was performed using a LightCycler® 480 (Roche) system. The following TaqMan^TM^ Assays containing the primer/probe sets of selected genes of interest were used: Hs00542476_g1 (*TNFSF14*), Hs01596929_m1 (*YOD1*), Hs00174469_m1 (*KLRB1*), Hs01087625_m1 (*CLEC4A*), Hs01592899_m1 (*SIGLEC14*). Expression data were normalized to Hs00187842_m1 (*B2M*). Data were normalized using an adapted protocol from [25].

*Assessment of robustness of gene expression measurement*

To obtain a robust signature, we only considered 6,966 transcripts, in which expression was significantly correlated (ρ≥0.5, FDR≤0.05) when measuring a subset of 72 samples with Next Generation Sequencing (*Suppl.Fig. 2D, E*). We also required a certain minimum absolute fold-change (1.1), significance (FDR≤0.5), expression level (expressed in ≥10% of cases or controls), robust uni- and multivariate associations (*i.e.* same effect size direction in uni- and multivariate models), high AUC-rank in predicting ICU admission or mortality with expression data from study inclusion in a leave-one-out cross validation scheme, and top-genes to overlap in at least 90% of the 240 cross-validation result lists (*Suppl.Fig. 1B*).

All gene expression levels from the expression chip data with expression levels from the RNA sequencing were correlated using spearman rank correlation. Transcripts that were robustly measurable with both techniques when correlation between chip data and sequencing data showed a statistically significant rank-correlation ρ≥0.5 and FDR≤5% rate were considered. This non-parametric method does not require comparable levels or distributions of data between the two platforms. It compares the ranks of each method. By employing spearman rank correlation, the results from both microarray and RNA sequencing were combined without the need for additional normalization steps to account for platform-specific biases.

**Identification of the predictive signature**

In order to identify a signature that could predict deterioration to severe CAP, we randomly separated study participants into a discovery and validation cohort. Transcriptome data from different days after inclusion were assessed for time series analysis. The signature was identified in a discovery cohort (N=240). Cross-validation was performed by creating 240 individual leave-one-out datasets, using the robustly measurable transcripts. For each of the datasets, we performed mixed effects modeling association with the same time-series regression model to utilize multiple time points per individual, resulting in 240 association lists. These lists were filtered to identify the most promising predictive transcripts (termed top-genes in *e-Fig.1* using the following steps. *a*) Transcripts with an expression below a minimum fold-change (FC) and above a minimal FDR were removed to create a top list for each model. Several cutoffs were tested for this purpose (fold-changes between 1.1–1.5 in steps of 0.01, maximum FDR of 0.05, 0.1, 0.2, or 0.5) [26]. We identified a fold-change of 1.33 and an FDR of 0.2 as the optimum. *b*) Overexpressed genes that were actually expressed in less than 10% of all cases, and down-regulated genes that were expressed in less than 10% of controls were filtered to remove genes with low expression level. *c*) Multiple transcripts from the same gene were filtered such that only unique genes remained in each top list. *d*) Genes highly correlated with the expression levels of other genes from the same top list (*i.e*. keeping only one of the genes when several genes had a Pearson-correlation higher than 0.8) were filtered. For step c and d, we favored transcripts with higher effect sizes (1-digit up-rounded), those not mapping at multiple locations in the human genome and higher expression levels to enrich robust findings and increase robustness. *e*) Genes that switched effect size direction upon including all other genes of the respective top list as covariates in association analysis were filtered to favor genes with consistent effects. This was done within each combination of cutoffs.

Next, 240 candidate signatures were determined on the basis of the steps a)-e) as previously described [27, 28]. Briefly, for each patient, the geometric mean of the log2-transformed expression levels of downregulated genes was subtracted from that of the upregulated genes. The geometric means are weighted by the number of down- and upregulated genes.

For each combination of fold-change cutoffs and FDR cutoffs (step a)), the predictive performance to predict the endpoint (quantified as the area under the curve (AUC) from the receiver operator characteristic (ROC) analysis) of the expression scores measured on the day of study inclusion was analyzed for each of the 240 left-out patients. The resulting AUCs were used to rank the parameter combination of FC and FDR considered in step a).

To unite each of the 240 gene lists, we identified genes that frequently appeared across multiple top lists. We tested different cutoffs for multiple appearances, i.e., genes that were present in 50%, 60%, 70%, 80% and 90% of all 240 top lists, using finally the 90% cut-off value. Expression scores for candidate-signatures were calculated from overlapping genes, using chip data measured from whole blood on the day of study inclusion. The predictive performance of these scores was evaluated on the basis of the AUC of the complete discovery cohort. The candidate signatures with an AUC > 0.8 were selected. The AUC from the scores combining the candidate-signatures and the SOFA score was used for the final ranking.

AUCs of ROCs were calculated with the R package pROC 1.18.0 [29]. Confidence intervals as well as comparisons of two ROC curves were calculated using 2,000 stratified bootstrap samples, p-values of single analyses were calculated based on the Wilcoxon-Mann-Whitney U-statistic. When combining two scores to analyze combined prediction, the unweighted sum of both scaled scores was utilized.

# SUPPLEMENTARY FIGURES

#
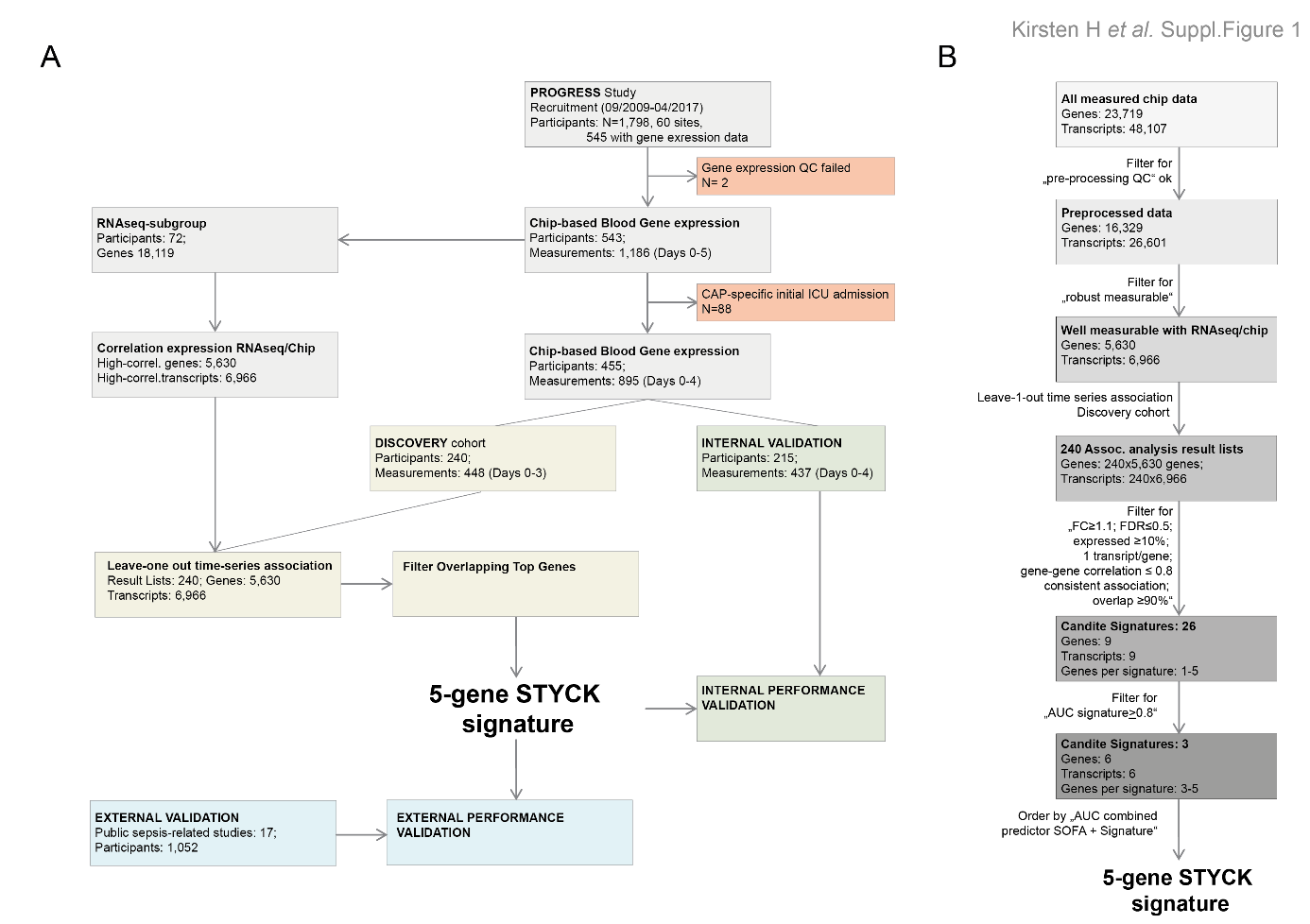


**e-Figure 1: (A) Study design and (B) flow chart for the identification and validation of the 5-gene STYCK signature.** Characterization of 1,798 recruited and 455 finally included participants is shown in *Table 1* and *Suppl.Table 2*.

Abbreviations: AUC.. area under the curve; FC.. fold change; FDR.. false discovery rate; QC.. quality control


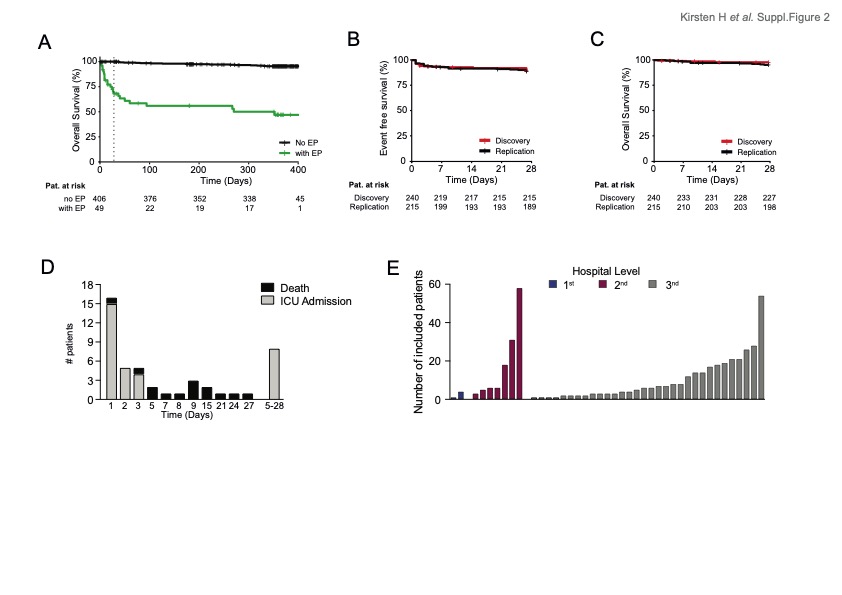


**e-Figure 2: (A)** Long-term survival of all patients with CAP. **(B)** Event-free 28-day survival and **(C)** 28-day survival of patients from the discovery and validation cohorts. EP indicates deterioration to ICU admission or death. (**D**) Time after study inclusion to either ICU-admission or death after first transcriptome measurement. (**E**) Inclusion of study participants per center and hospital level, using hospital level definitions as previously described [30].

Abbreviations: Pat.. patients.

**
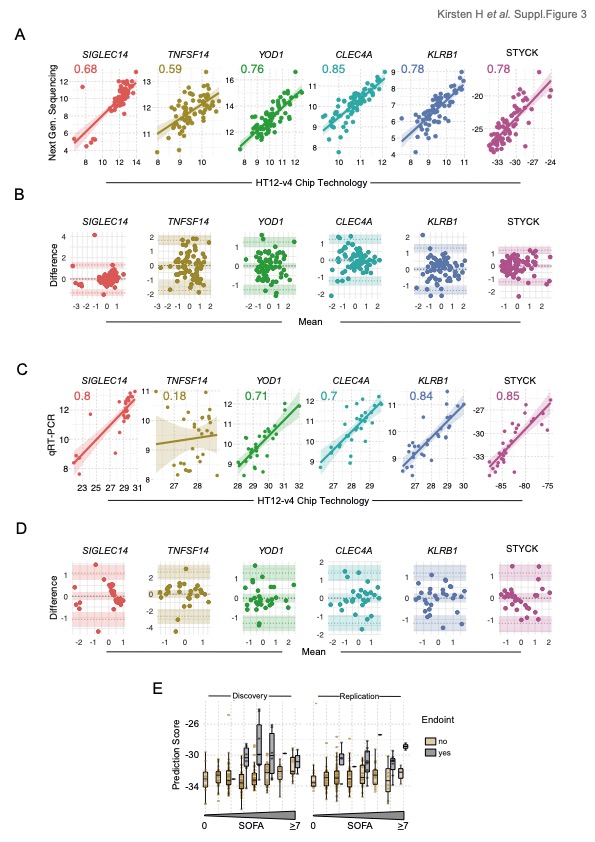
**

**e-Figure 3:** **(A)** Spearman-Correlation of gene expression of the five transcripts individually and the STYCK signature measured with HT12-v4 Chip *vs.* NGS. **(B)** Bland-Altman analysis assessing the agreement between HT12v4 chip and NGS. **(C)** Spearman-Correlation of gene expression of the five transcripts individually and the STYCK signature measured with HT12-v4 Chip *vs* qRT-PCR. **(D)** Bland-Altman analysis assessing the agreement between HT12v4 chip and qRT-PCR. The Spearman plots contain the Spearman correlation coefficient (ρ) and the line of best linear fit and 95% confidence interval are displayed. The Bland-Altman plots contain the mean difference (bias) ±1.96 standard deviations and the respective 95% confidence intervals are shown. **(E)** Predictive performance of the signature for patients stratified by present disease severity (SOFA) at the time of study inclusion. Different levels of the STYCK signature for cases reaching the endpoint later on compared with controls are also present for patient with initially lower SOFA values.

Abbreviations: NGS.. Next Generation Sequencing; SOFA.. Sequential Organ Failure Assessment.


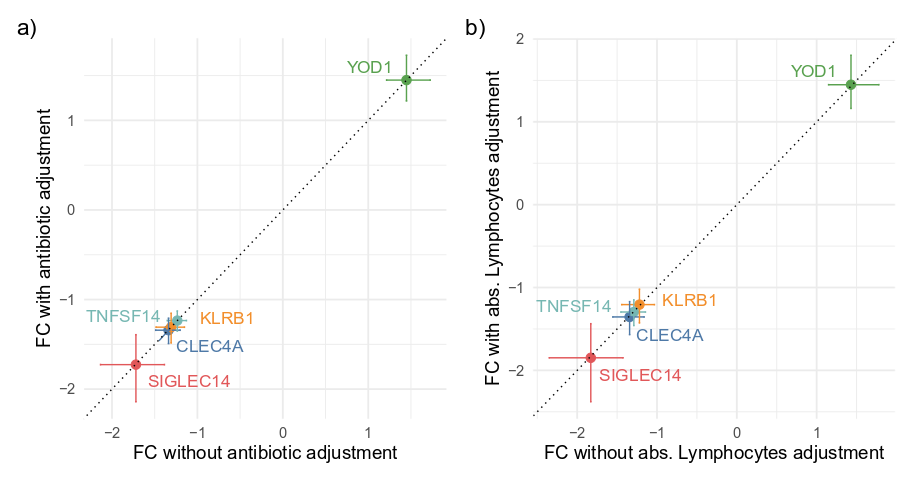


**e-Figure 4:** Sensitivity analyses evaluating the impact of pre-admission antibiotic use and absolute lymphocyte counts on gene signature effect sizes. The scatter plots compare the fold-changes (FC) and 95% confidence intervals for the five signature genes from mixed-effects models with and without additional covariates. The dotted line represents the line of identity. **(a)** The model adjusted for antibiotic use within 5 days prior to admission (y-axis) is compared to the primary model (x-axis) using 873 observations from 445 patients. **(b)** The model adjusted for the absolute lymphocyte count (y-axis) is compared to the primary model (x-axis) using a subset of 603 observations from 317 patients with available data. The dotted line indicates perfect concordance. In both analyses, the very high concordance demonstrates the signature's robustness to these potential confounders.


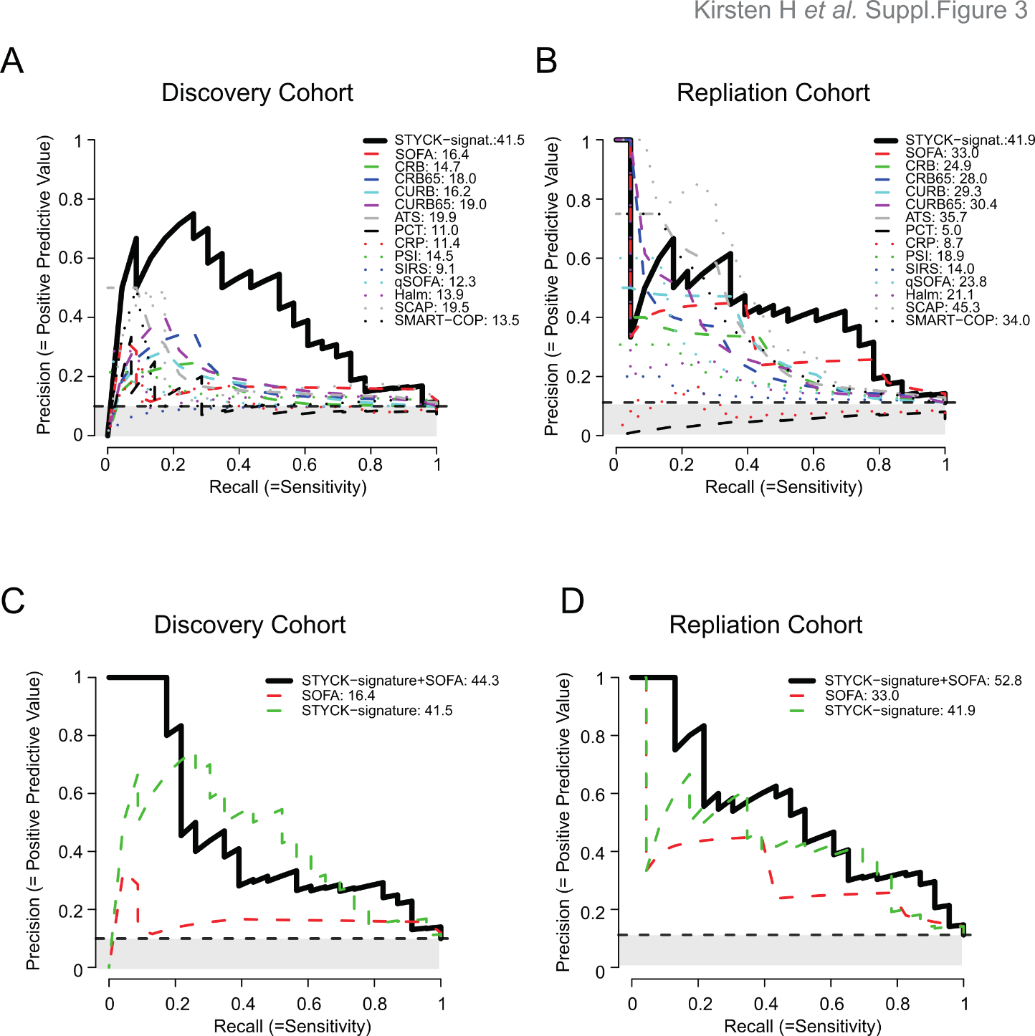


**e-Figure 5:** Predictive performance of the STYCK signature – area under the precision-recall curve (AUC‑PR) **A, B** Comparison to clinical scores and serologic markers in (**A**) the discovery cohort and (**B**) the validation cohort. Details on clinical scores are reported in [2]. (**C, D**) Predictive performance in combining the STYCK signature with the SOFA score in (**C**) the discovery cohort and (**D**) the validation cohort. Values represent AUC-PR

Abbreviations: ATS.. American Thoracic Society minor criteria; AUC.. area under the curve; CRB: similar as CURB without blood-urea nitrogen; CURB65, CRB65: as CURB, CRB but additionally considering age ≥65; CRP.. C-Reactive Protein; CURB.. Confusion, Urea, Respiratory rate, Blood pressure score; PCT.. Procalcitonin level; PSI.. Pneumonia Severity Index (Fine score); qSOFA.. quick SOFA score; Halm.. Halm score; SCAP.. Severe CAP score; SIRS.. Systemic Inflammatory Response Syndrome; SMART-COP.. systolic blood pressure, multilobar chest radiography involvement, low albumin level, high respiratory rate, tachycardia, confusion, poor oxygenation, and low arterial pH score; SOFA.. Sequential Organ Failure Assessment.


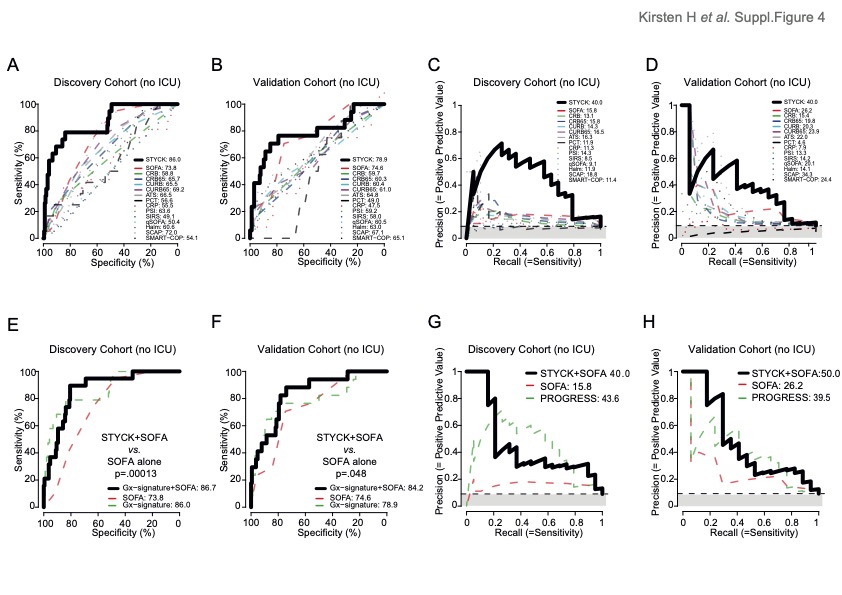


**e-Figure 6:** Predictive performance of the STYCK signature – Sensitivity analysis in which CAP patients with non-respiratory indications for ICU admission were excluded**. A, B** Comparison to clinical scores and serologic markers in (**A**) the discovery cohort and (**B**) the validation cohort. Details on clinical scores are reported in [2]. (**C, D**) Same data shown as precision-recall curves. **E-H** Predictive performance in combining the STYCK signature with the SOFA score in (**E**) the discovery cohort and (**F**) the validation cohort. Values are the area under the receiver operator characteristic. (**G, H**) Same data shown as precision-recall curves. P-values were calculated using 2,000 bootstrap-samples.

Abbreviations: ATS.. American Thoracic Society minor criteria; AUC.. area under the curve; CRB: similar as CURB without blood-urea nitrogen; CURB65, CRB65: as CURB, CRB but additionally considering age ≥65; CRP.. C-Reactive Protein; CURB.. Confusion, Urea, Respiratory rate, Blood pressure score; PCT.. Procalcitonin level; PSI.. Pneumonia Severity Index (Fine score); qSOFA.. quick SOFA score; Halm.. Halm score; SCAP.. Severe CAP score; SIRS.. Systemic Inflammatory Response Syndrome; SMART-COP.. systolic blood pressure, multilobar chest radiography involvement, low albumin level, high respiratory rate, tachycardia, confusion, poor oxygenation, and low arterial pH score; SOFA.. Sequential Organ Failure Assessment.

**
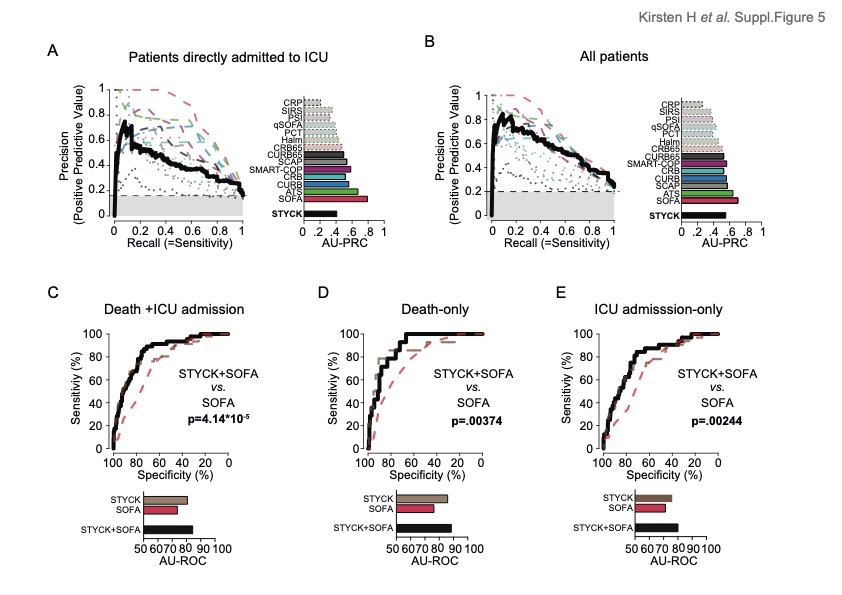
**

**e-Figure 7:** Predictive performance of the STYCK score quantified as precision-recall curves (PRC) in (**A**) Patients directly admitted to the ICU and (**B**) all patients with CAP. Comparison of prediction performances of the STYCK signature to 14 clinical scores or parameters quantified as AUC of the PRC curve (Bar plot). (**C**) AUC-ROC curves for the performance of the STYCK signature, SOFA score, and combined STYCK-SOFA predictor in discriminating patients in the discovery and validation cohorts with (**C**) both death and ICU admission, (**D**) death only or (**E**) ICU-admission only.

Abbreviations: ATS.. American Thoracic Society minor criteria; AUC.. area under the curve; CRB: similar as CURB without blood-urea nitrogen; CURB65, CRB65: as CURB, CRB but additionally considering age ≥65; CRP.. C-Reactive Protein; CURB.. Confusion, Urea, Respiratory rate, Blood pressure score; PCT.. Procalcitonin level; PSI.. Pneumonia Severity Index (Fine score); qSOFA.. quick SOFA score; Halm.. Halm score; SCAP.. Severe CAP score; SIRS.. Systemic Inflammatory Response Syndrome; SMART-COP.. systolic blood pressure, multilobar chest radiography involvement, low albumin level, high respiratory rate, tachycardia, confusion, poor oxygenation, and low arterial pH score; SOFA.. Sequential Organ Failure Assessment.

**SUPPLEMENTARY TABLES**

**e-Table 1**: List of study centers contributing patients to the PROGRESS study between 09/2009 and 04/2017.

**e-Table 2***:* Baseline characteristics and outcomes of all 1,798 patient participants recruited in the PROGRESS cohort at the time of this study.

Abbreviations: CAP.. community-acquired pneumonia; COPD.. chronic obstructive pulmonary disease; HAP.. hospital-acquired pneumonia; ICU.. intensive care unit; IQR.. interquartile range; SOFA.. Sequential Organ Failure Assessment.

**e-Table 3**: Distribution of timepoints with measured gene expression data for the discovery and validation cohort. p-value corresponds to testing similarity of the distribution of measurements when comparing the discovery and validation cohort (Fisher`s exact test).

**e-Table 4:** Association between a severe CAP course and gene expression levels in the discovery cohort. Results are shown with and without adjustment for the SOFA score. Measurements numbers are larger than number of patients since repeated measurements were included when available.

Abbreviations: FDR.. false discovery rate.

**e-Table 5**: Full list of all associations between gene expression and reaching the endpoint adjusted for SOFA score.

Associations were analyzed in a mixed model accounting for repeated measurements of patients by modeling patient ID as a random intercept, as implemented in the R package limma [21]. Additionally, analyses were adjusted for the SOFA score determined at presentation, age, sex, percent lymphocytes, percent neutrophil granulocytes, log BMI, the modified Charlson comorbidity score, and smoking in the last 12 months.

Abbreviations: BMI.. body mass index; cEP.. composite endpoint.

**e-Table 6**: Full list of pathways enriched for genes associated with reaching the endpoint and their activation status (Discovery Cohort)

**e-Table 7**: Full list of all associations between gene expression and reaching the endpoint not adjusted for SOFA score.

Associations were analyzed in a mixed model accounting for repeated measurements of patients by modeling patient ID as a random intercept, as implemented in the R package limma [21]. Additionally, analyses were adjusted for age, sex, percent lymphocytes, percent neutrophil granulocytes, log BMI, comorbidity, and smoking in the last 12 months.

Abbreviations: BMI.. body mass index.

**e-Table 8:** Information about the role of STYCK genes. Information on the aliases is from https://www.genecards.org/.

**e-Table 9:** Association of signature genes in the discovery and validation cohort. Regression model of the association of each gene adjusted on age, sex, comorbidity, cell counts, smoking, SOFA-score and body mass index. AUC quantifies prediction accuracy of severe CAP using expression data from day of study inclusion, only. The FDR accounts for multiple testing of all genes of the discovery panel and the 5 tested genes in the validation panel.

Abbreviations: AUC.. area under the curve; FC.. fold change; FDR.. false discovery rate; HGNC.. Human Genome Organization Gene Nomenclature Committee (https://www.genenames.org)."

**e-Table 10:** Predictive performance of subsets of the STYCK signature including 1, 2, 3, or 4 of the 5 signature genes

Abbreviations: AUC.. area under the curve; ROC.. receiver operated characteristics

**e-Table 11**: Performance of the PROGRESS gene expression signature predicting mortality in public sepsis-related datasets.

Abbreviations: AUC, area under the curve.

**e-Table 12**: Transcripts correlating with the five genes of the STYCK signature in the expression data of the discovery and validation cohort.

Abbreviations: FDR.. false discovery rate.

**REFERENCES SUPPLEMENT**

REfer

1. Ahnert P, Creutz P, Scholz M, Schütte H, Engel C, Hossain H, Chakraborty T, Bauer M, Kiehntopf M, Völker U, Hammerschmidt S, Loeffler M, Suttorp N, (2016) PROGRESS - prospective observational study on hospitalized community acquired pneumonia. BMC pulmonary medicine 16: 108

2. Ahnert P, Creutz P, Horn K, Schwarzenberger F, Kiehntopf M, Hossain H, Bauer M, Brunkhorst FM, Reinhart K, Völker U, Chakraborty T, Witzenrath M, Löffler M, Suttorp N, Scholz M, (2019) Sequential organ failure assessment score is an excellent operationalization of disease severity of adult patients with hospitalized community acquired pneumonia - results from the prospective observational PROGRESS study. Critical care (London, England) 23: 110

3. Charlson ME, Pompei P, Ales KL, MacKenzie CR, (1987) A new method of classifying prognostic comorbidity in longitudinal studies: development and validation. J Chronic Dis 40: 373-383

4. Fine MJ, Singer DE, Hanusa BH, Lave JR, Kapoor WN, (1993) Validation of a pneumonia prognostic index using the MedisGroups Comparative Hospital Database. Am J Med 94: 153-159

5. Products CfHM (2016) ICH E6 (R2) Good clinical practice: Step 5. In: Editor (ed)^(eds) Book ICH E6 (R2) Good clinical practice: Step 5. European Medicines Agency, City, pp. 68

6. Declaration.of.Helsinki (2018) WMA Declaration of Helsinki – Ethical Principles for Medical Research Involving Human Subjects – WMA – The World Medical Association. In: Editor (ed)^(eds) Book WMA Declaration of Helsinki – Ethical Principles for Medical Research Involving Human Subjects – WMA – The World Medical Association. World Medical Association, Inc., City, pp. 4

7. Du P, Kibbe WA, Lin SM, (2008) lumi: a pipeline for processing Illumina microarray. Bioinformatics 24: 1547-1548

8. Johnson WE, Li C, Rabinovic A, (2007) Adjusting batch effects in microarray expression data using empirical Bayes methods. Biostatistics 8: 118-127

9. Cohen Freue GV, Hollander Z, Shen E, Zamar RH, Balshaw R, Scherer A, McManus B, Keown P, McMaster WR, Ng RT, (2007) MDQC: a new quality assessment method for microarrays based on quality control reports. Bioinformatics (Oxford, England) 23: 3162–3169

10. Kampf C, Specht M, Scholz A, Puppel SH, Doose G, Reiche K, Schor J, Hackermuller J, (2019) uap: reproducible and robust HTS data analysis. BMC Bioinformatics 20: 664

11. Schubert M, Lindgreen S, Orlando L, (2016) AdapterRemoval v2: rapid adapter trimming, identification, and read merging. BMC Res Notes 9: 88

12. Bray NL, Pimentel H, Melsted P, Pachter L, (2016) Near-optimal probabilistic RNA-seq quantification. Nat Biotechnol 34: 525-527

13. Soneson C, Love MI, Robinson MD, (2015) Differential analyses for RNA-seq: transcript-level estimates improve gene-level inferences. F1000Res 4: 1521

14. Daley T, Smith AD, (2013) Predicting the molecular complexity of sequencing libraries. Nat Methods 10: 325-327

15. Kim D, Paggi JM, Park C, Bennett C, Salzberg SL, (2019) Graph-based genome alignment and genotyping with HISAT2 and HISAT-genotype. Nat Biotechnol 37: 907-915

16. Wang L, Wang S, Li W, (2012) RSeQC: quality control of RNA-seq experiments. Bioinformatics 28: 2184-2185

17. Ewels P, Magnusson M, Lundin S, Kaller M, (2016) MultiQC: summarize analysis results for multiple tools and samples in a single report. Bioinformatics 32: 3047-3048

18. Wingett SW, Andrews S, (2018) FastQ Screen: A tool for multi-genome mapping and quality control. F1000Res 7: 1338

19. Langmead B, Salzberg SL, (2012) Fast gapped-read alignment with Bowtie 2. Nat Methods 9: 357-359

20. Love MI, Huber W, Anders S, (2014) Moderated estimation of fold change and dispersion for RNA-seq data with DESeq2. Genome Biol 15: 550-550

21. Ritchie ME, Phipson B, Wu D, Hu Y, Law CW, Shi W, Smyth GK, (2015) limma powers differential expression analyses for RNA-sequencing and microarray studies. Nucleic acids research 43: e47

22. Newman AM, Liu CL, Green MR, Gentles AJ, Feng W, Xu Y, Hoang CD, Diehn M, Alizadeh AA, (2015) Robust enumeration of cell subsets from tissue expression profiles. Nature methods 12: 453–457

23. Strimmer K, (2008) fdrtool: a versatile R package for estimating local and tail area-based false discovery rates. Bioinformatics (Oxford, England) 24: 1461–1462

24. Krämer A, Green J, Pollard J, Tugendreich S, (2014) Causal analysis approaches in Ingenuity Pathway Analysis. Bioinformatics (Oxford, England) 30: 523–530

25. Livak KJ, Schmittgen TD, (2001) Analysis of relative gene expression data using real-time quantitative PCR and the 2(-Delta Delta C(T)) Method. Methods 25: 402-408

26. Sweeney TE, Haynes WA, Vallania F, Ioannidis JP, Khatri P, (2017) Methods to increase reproducibility in differential gene expression via meta-analysis. Nucleic Acids Res 45: e1

27. Sweeney TE, Shidham A, Wong HR, Khatri P, (2015) A comprehensive time-course-based multicohort analysis of sepsis and sterile inflammation reveals a robust diagnostic gene set. Sci Transl Med 7: 287ra271

28. Sweeney TE, Wong HR, Khatri P, (2016) Robust classification of bacterial and viral infections via integrated host gene expression diagnostics. Science translational medicine 8: 346ra391

29. Robin X, Turck N, Hainard A, Tiberti N, Lisacek F, Sanchez J-C, Müller M, (2011) pROC: an open-source package for R and S+ to analyze and compare ROC curves. BMC bioinformatics 12: 77

30. Mulligan J, Fox-Rushby JA, Adams T, Johns B, Mills A, (2005) Unit Costs of Health Care Inputs in Low and Middle Income Regions. Working Paper 9, Disease Control Priorities Fogarty International Center, National Institutes of Health, Bethesda, MD September 2003

(revised June 2005)
